# Supplementary material for: Prioritizing Parkinson’s disease genes using population-scale transcriptomic data
Source: Nat Commun. 2019 Mar 1;10:994. doi: 10.1038/s41467-019-08912-9 (PMC6397174; doi:10.1038/s41467-019-08912-9)
Supplement: Supplementary file 1 — Supplementary Information [file 41467_2019_8912_MOESM1_ESM.pdf]

Supplementary Tables and Figures

Prioritizing Parkinson's Disease genes using population-scale transcriptomic data

Yang I Li, Garrett Wong, Jack Humphrey, and Towfique Raj

Supplementary Table 1: Summary of replication of DLPFC TWAS results.

Data Source	Gene	Splicing Event	CHR	ROSMAP.TWAS.Z	ROSMAP.T	CMC.TWAS.Z
ROSMAP_DLFPC_expression	CTSB		8	-4.89	1.01E-06	-5.74
ROSMAP_DLFPC_expression	RAB4A		1	-2.16	0.03084	-4.11
ROSMAP_DLFPC_expression	RAB7L1		1	3.71	2.08E-04	4.34
ROSMAP_DLFPC_expression	TMEM163		2	3.58	3.49E-04	4.80
ROSMAP_DLFPC_expression	CD38		4	-5.29	1.23E-07	-4.56
ROSMAP_DLFPC_expression	MMRN1		4	3.89	9.84E-05	6.52
ROSMAP_DLFPC_expression	GPNMB		7	3.78	1.57E-04	5.50
ROSMAP_DLFPC_expression	ATG14		14	-2.02	4.31E-02	-3.61
ROSMAP_DLFPC_expression	HSD3B7		16	-2.15	3.18E-02	-4.24
ROSMAP_DLFPC_expression	PDLIM2		8	-1.88	5.02E-02	-3.92
ROSMAP_DLFPC_expression	NUDT14		14	2.29	2.21E-02	3.51
ROSMAP_DLFPC_splicing	CAMLG	5:134074482:134079677	5	-3.38	0.00073	-3.69
ROSMAP_DLFPC_splicing	PSD	10:104163742:104164340	10	1.96	5.06E-02	3.52
ROSMAP_DLFPC_splicing	RIMS2	8:105001649:105026734	8	-2.04	4.11E-02	3.33
ROSMAP_DLFPC_splicing	SNCA	4:90757680:90757894	4	6.43	1.30E-10	8.24
ROSMAP_DLFPC_splicing	TMEM175	4:949678:951612	4	2.73	6.38E-03	6.08
ROSMAP_DLFPC_splicing	MAPT	17:44049311:44055741	17	7.27	3.67E-13	10.17
ROSMAP_DLFPC_splicing	NT5C2	10:104934739:104952993	10	-2.32	2.03E-02	3.44
ROSMAP_DLFPC_splicing	ZRANB3	2:136148390:136261899	2	2.49	1.29E-02	4.40
ROSMAP_DLFPC_splicing	FAM47E	4:77192921:77204534	4	3.50	4.63E-04	3.44
ROSMAP_DLFPC_splicing	MGA	15:41952723:41988273	15	-3.11	1.85E-03	-3.38

Supplementary Table 2: MAPT TWAS Joint and Conditional Analysis summary.

ID	CHR	P0	P1	HSQ	BEST.GWAS.ID	BEST.GWAS.Z	EQTL.ID	EQTL.R2	EQTL.Z	EQTL.GW	NSNP	NWGT	MODEL	MODELCV.R2	MODELCV.PV	TWAS.Z	TWAS.P	Joint TWAS.P	Dropped
CRHR1	17	43861645	43913191	0.04	rs17763086	-10.21	rs17689471	4.56E-02	5.23	-10.1749	31	16	enet	0.051276	8.75E-07	-9.46165	3.03E-21	8.60E-01	Y
CRHR1	17	43861645	43913191	0.0184	rs17763086	-10.21	rs17689471	5.31E-03	-3.28	-10.1749	69	69	blup	0.013862	7.51E-03	8.37382	5.58E-17	1.30E-01	Y
MAPT	17	43971747	44105699	0.0334	rs17763086	-10.21	rs17689471	5.87E-02	-5.48	-10.1749	30	12	lasso	0.051245	8.82E-07	10.1749	2.57E-24	2.60E-24	
MAPT	17	43971747	44105699	0.0594	rs17763086	-10.21	rs17689471	1.10E-01	7.19	-10.1749	30	10	lasso	0.105666	1.39E-12	-10.1667	2.79E-24	8.80E-01	Y
MAPT	17	43971747	44105699	0.0622	rs17763086	-10.21	rs17689471	9.62E-02	6.83	-10.1749	29	29	blup	0.098858	7.66E-12	-10.1501	3.31E-24	1.90E-01	Y
LRRC37A2	17	44590075	44633014	0.1564	rs199451	-10.12	rs199448	1.97E-01	9.51	-10.0677	134	35	enet	0.200168	2.08E-23	-9.55776	1.20E-21	2.30E-01	Y

Supplementary Table 3: Sources of data for LDSC-SEG, GARFIELD, and TWAS analyses

**dataset**	**tissue**	**resources**	**N**	**citation**	**doi**
CMC	DLPFC	RNA-seq	537	Fromer et al. 2016	10.1038/nn.4399
ROSMAP	DLPFC	RNA-seq, methylation, H3K9AC	540	Ng et al. 2017	10.1038/nn.4632
immvar	monocyte	microarray expression, methylation, histone acetylation	211	Raj et al. 2014	10.1126/science.1249547
	monocyte	RNA-seq, methylation, H3K27AC, H3K4ME1	162-196		
blueprint	neutrophil	RNA-seq, methylation, H3K27AC, H3K4ME1	173-197	Chen et al. 2016	10.1016/j.cell.2016.10.026
	T-cell	RNA-seq, methylation, H3K27AC, H3K4ME1	104-169		
fairfax	monocyte	microarray expression	432	Fairfax et al. 2011	10.1126/science.1246949
cardiogenics	monocyte	microarray expression	802	Garnier et al. 2013; R	10.1371/journal.pgen.1002367, 10.1371/journal.pgen.1003240
IPDGC		GWAS	9581 cases, 33245 controls	Nalls et al. 2014	10.1038/ng.3043
23andMe		GWAS	4124 cases, 62037 controls	Nalls et al. 2014	10.1038/ng.3043

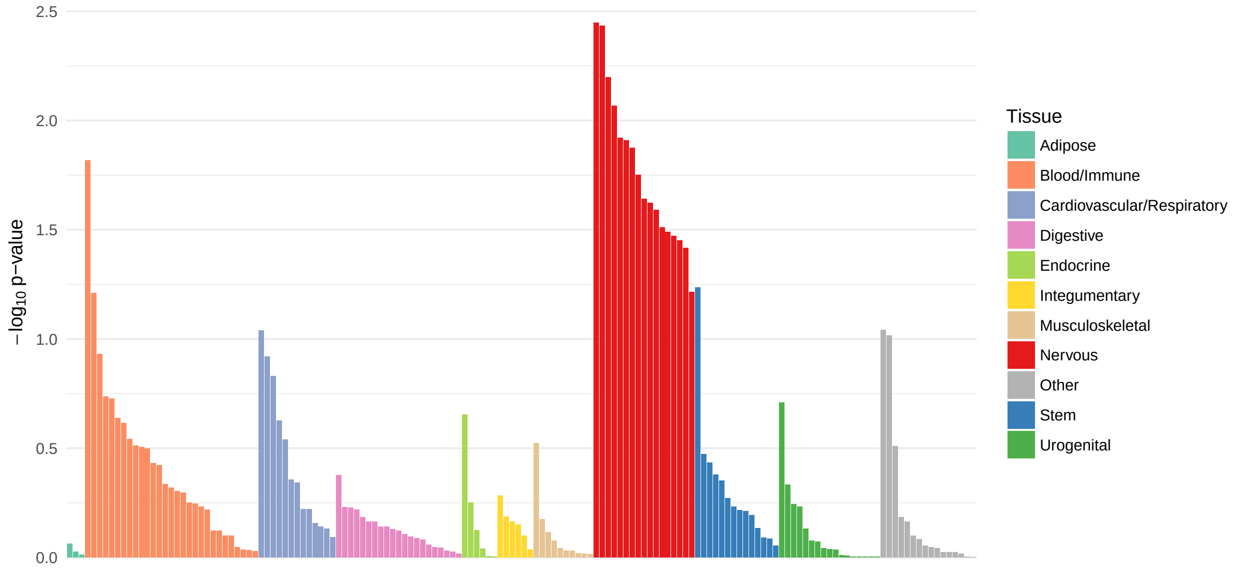

Supplementary Figure 1: LDscore-SEG analysis of PD GWAS enrichment in an atlas of 152 tissues from Pers *et al.*

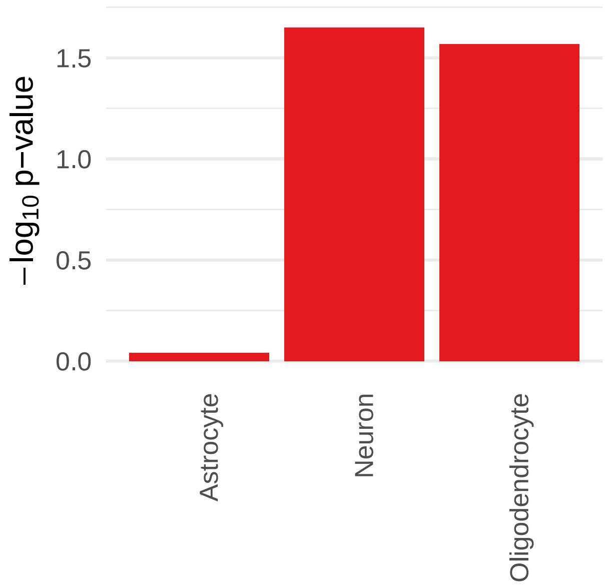

Supplementary Figure 2: LDscore-SEG analysis of PD GWAS enrichment in mouse CNS cells from Cahoy *et al.*

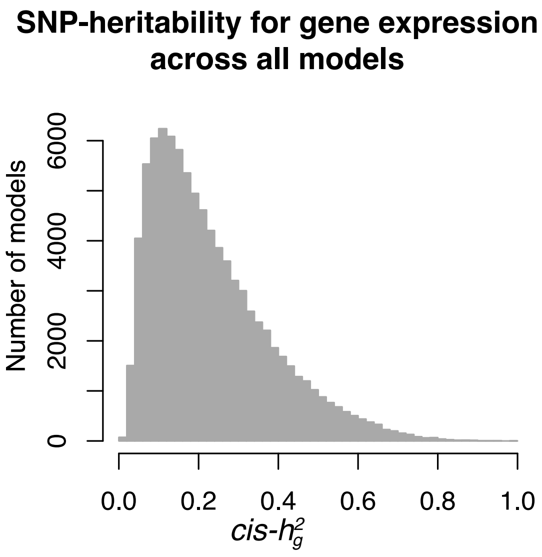

Supplementary Figure 3: SNP-heritability (cis) for gene expression across all models.

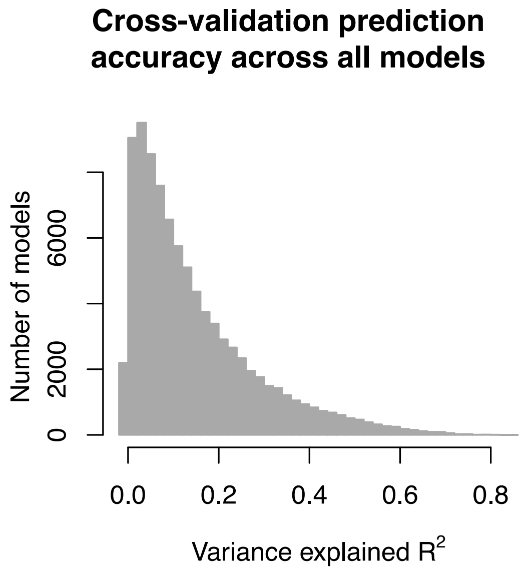

Supplementary Figure 4: Cross-validation prediction accuracy across all models.

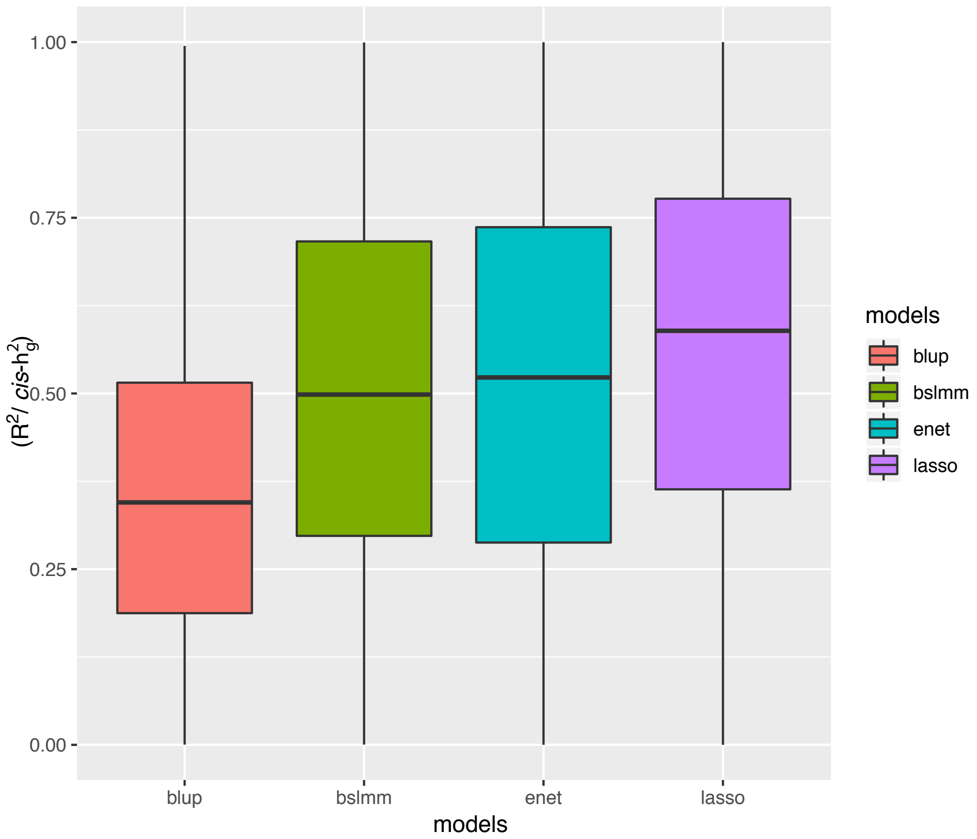

Supplementary Figure 5: Normalized prediction accuracy across four linear models (Elastic Net, LASSO, BLUP, and BSLMM). The model with the best cross-validation prediction accuracy (P ¡ 0.05) was used for prediction into the GWAS cohort.

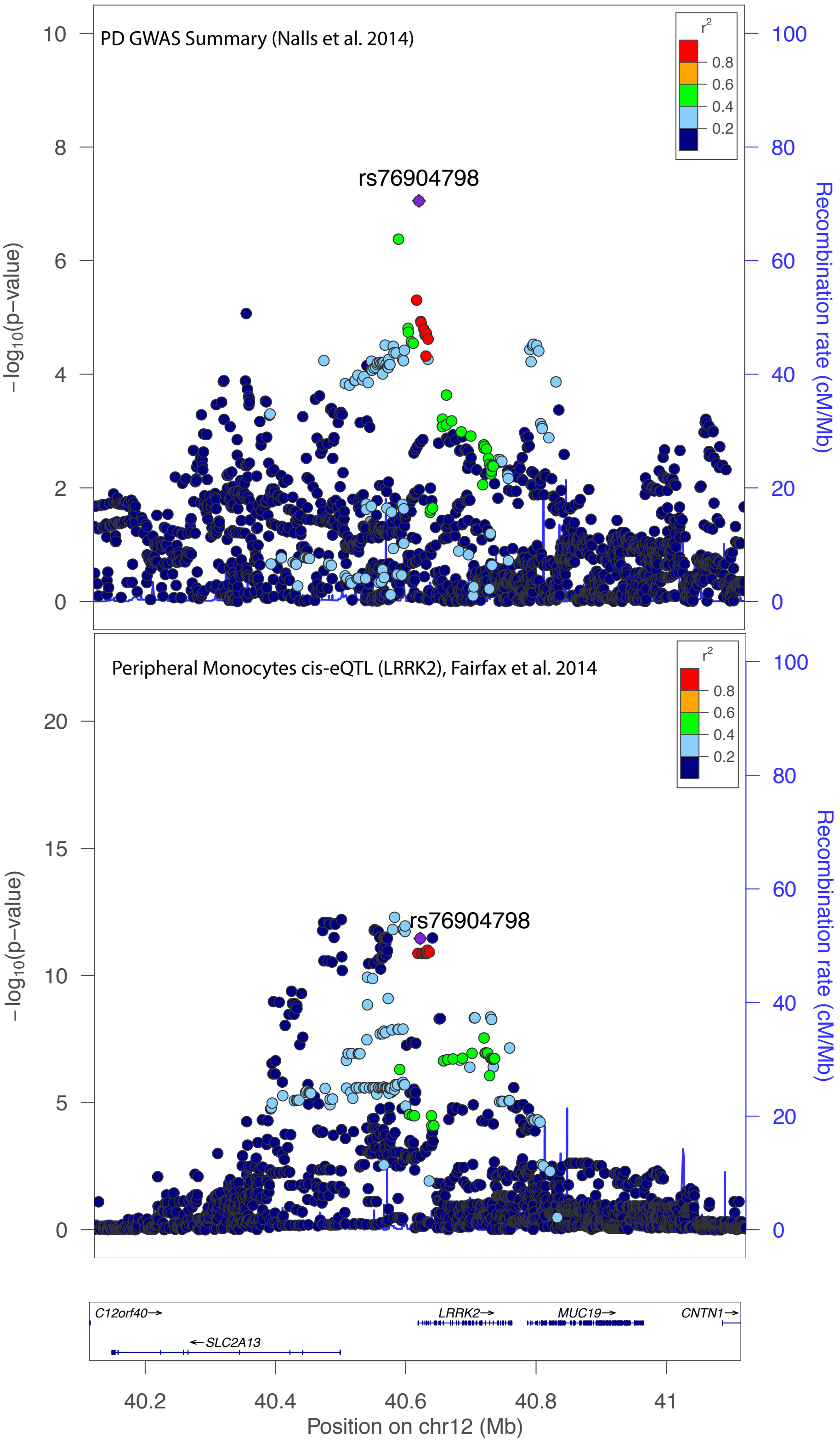

Supplementary Figure 6: Locuszoom plot of LRRK2 locus shows colocalization of primary monocyte eQTL and Parkinson's disease GWAS associations.

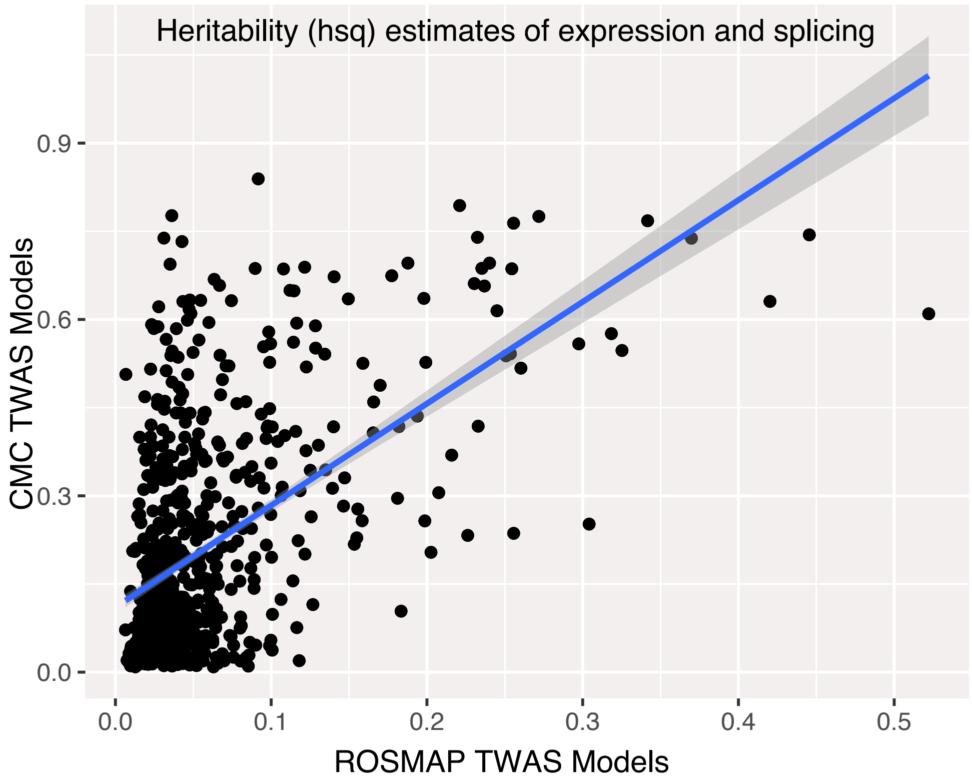

Supplementary Figure 7: CMC vs ROSMAP HSQ.

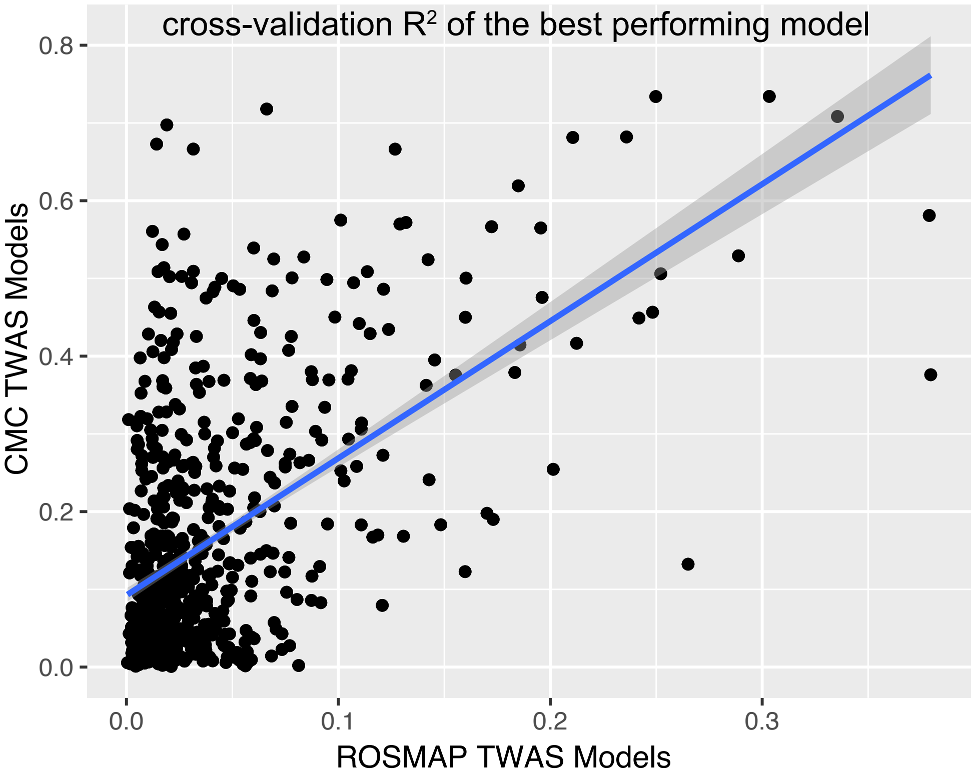

Supplementary Figure 8: CMC vs ROSMAP R^2.

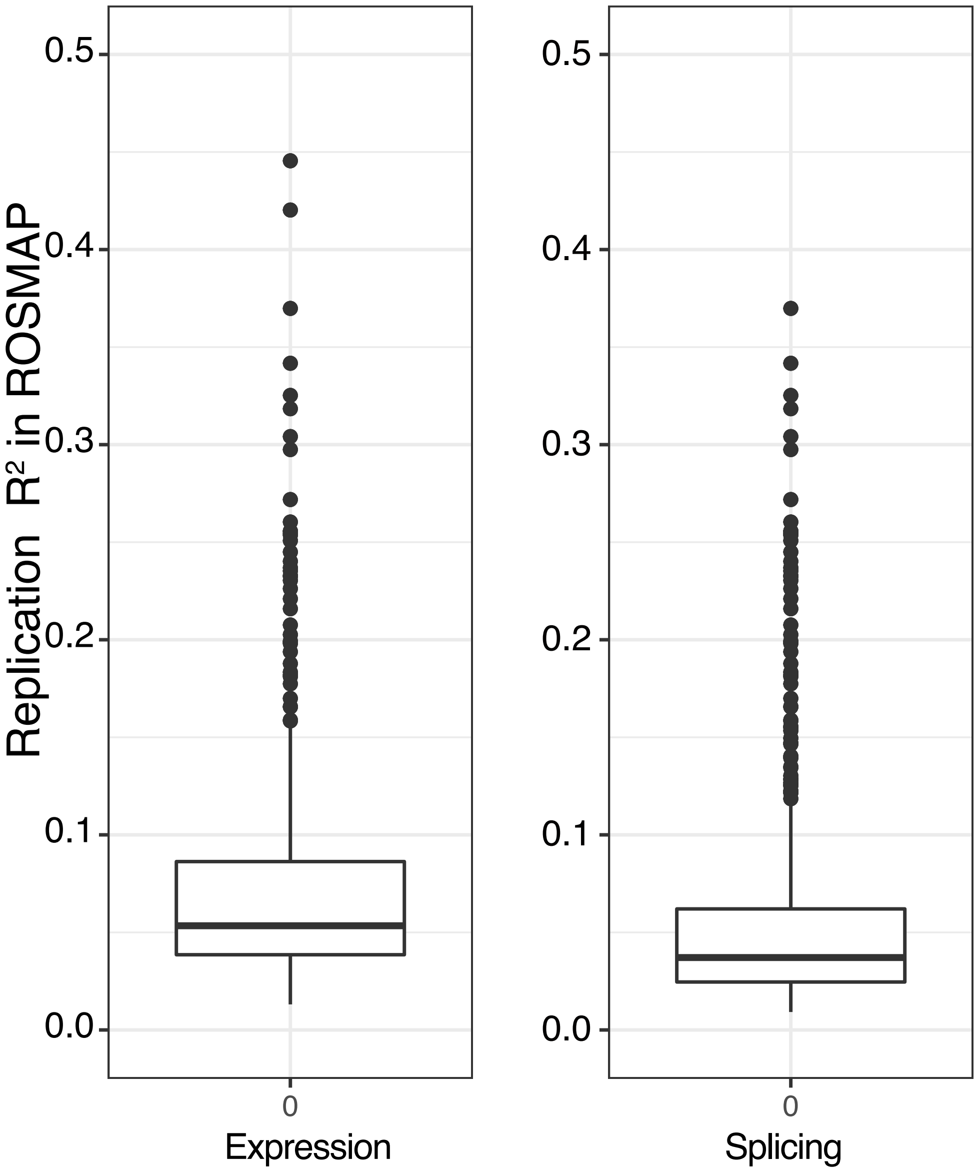

Supplementary Figure 9: CMC ROSMAP R^2 Boxplot.

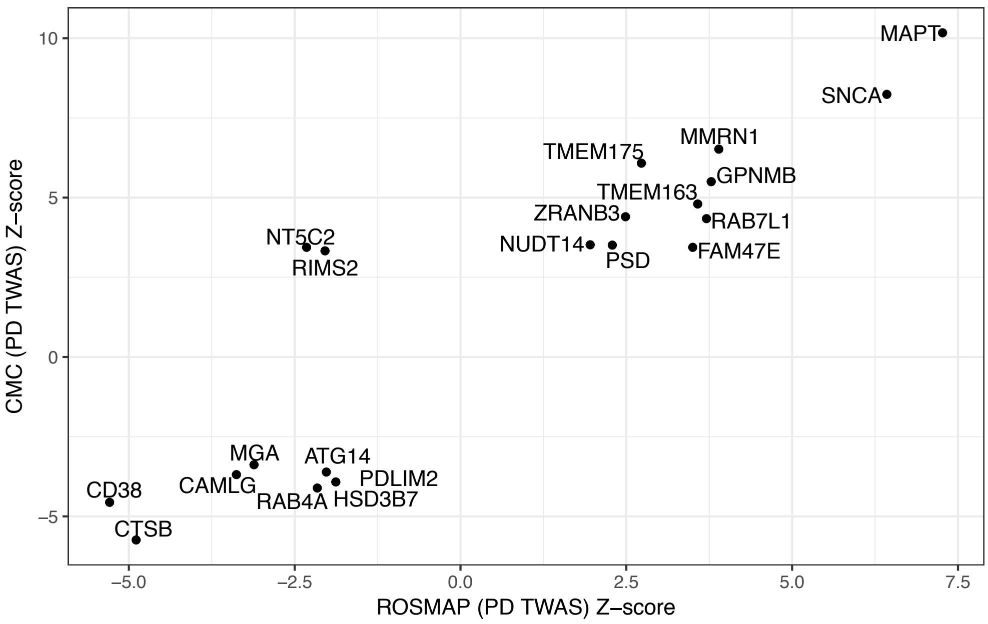

Supplementary Figure 10: Scatterplot of FDR < 0.05 TWAS Z-scores for ROS/MAP and CommondMind Consortium (CMC) DLPFC datasets shows concordant direction of effect.

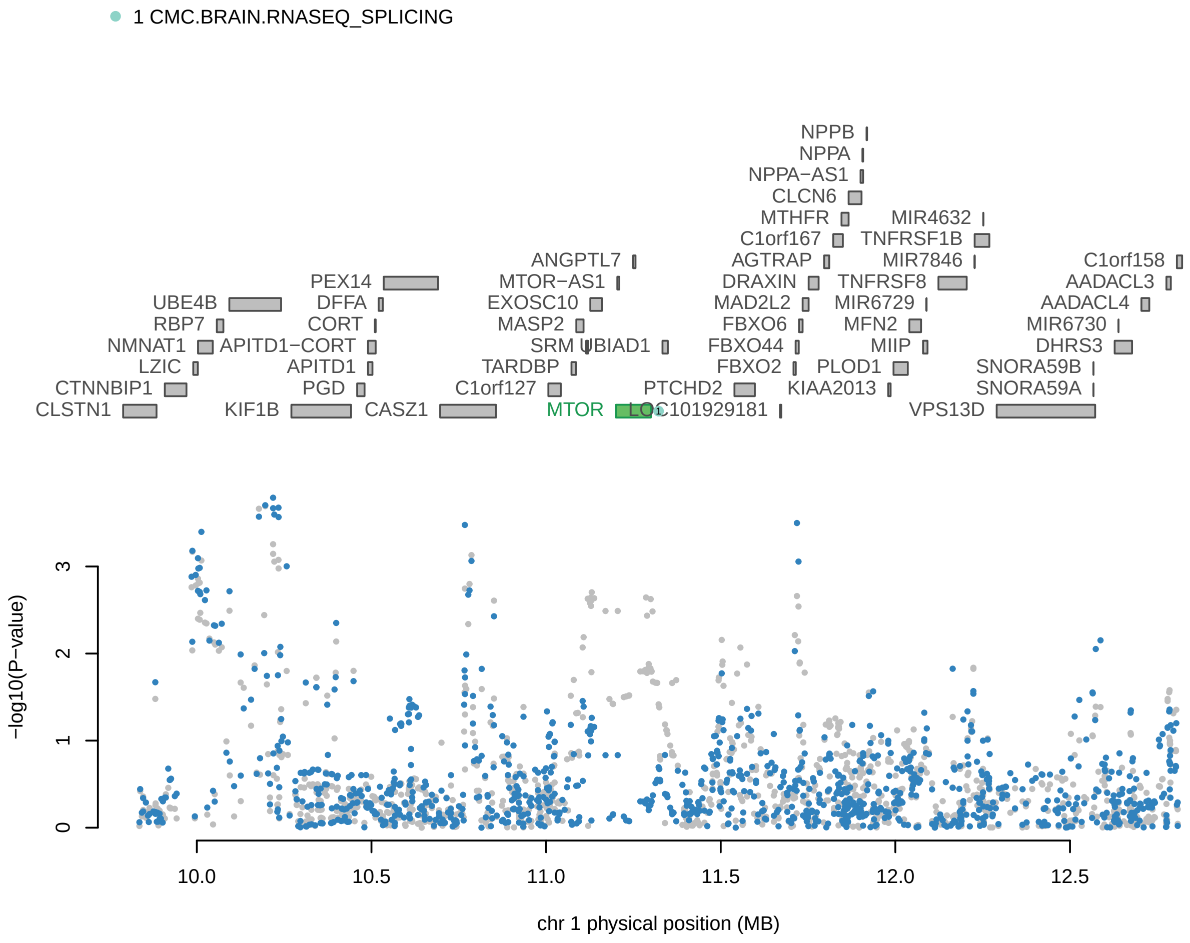

Supplementary Figure 11: FUSION plot of PD TWAS locus

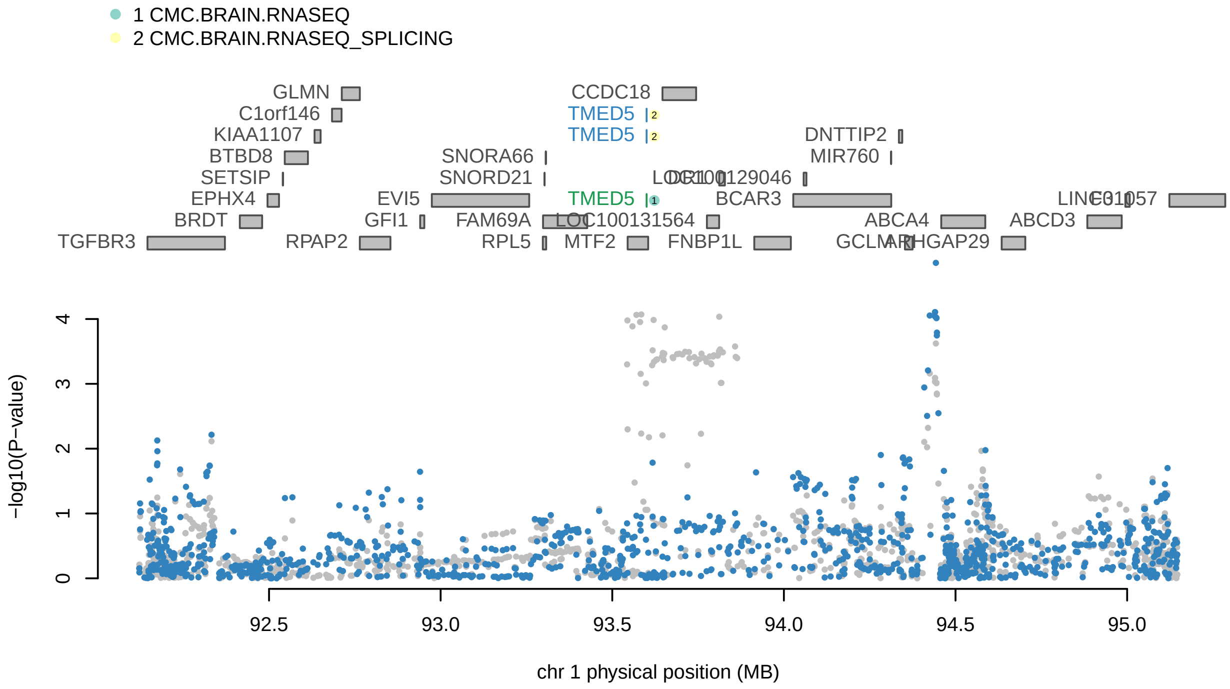

Supplementary Figure 12: FUSION plot of PD TWAS locus

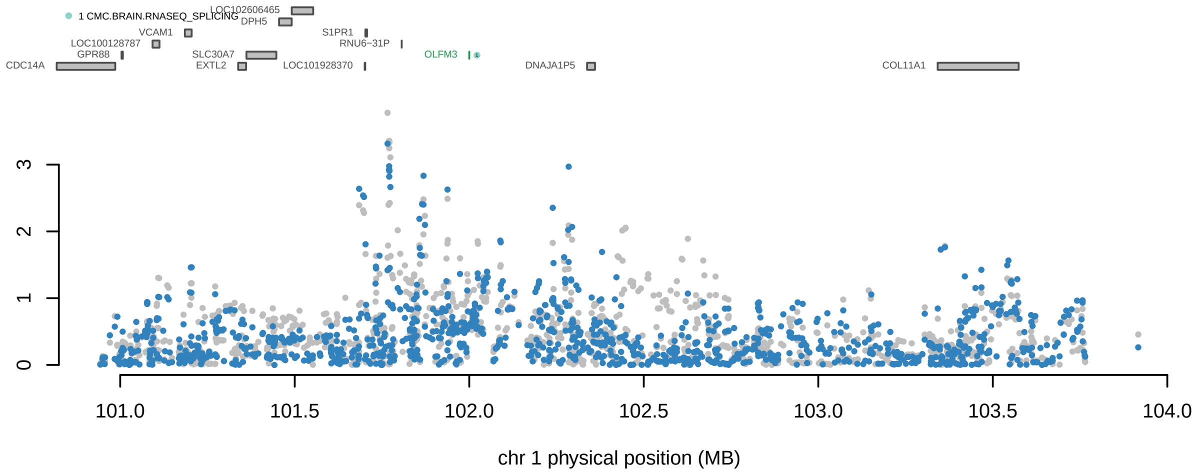

Supplementary Figure 13: FUSION plot of PD TWAS locus

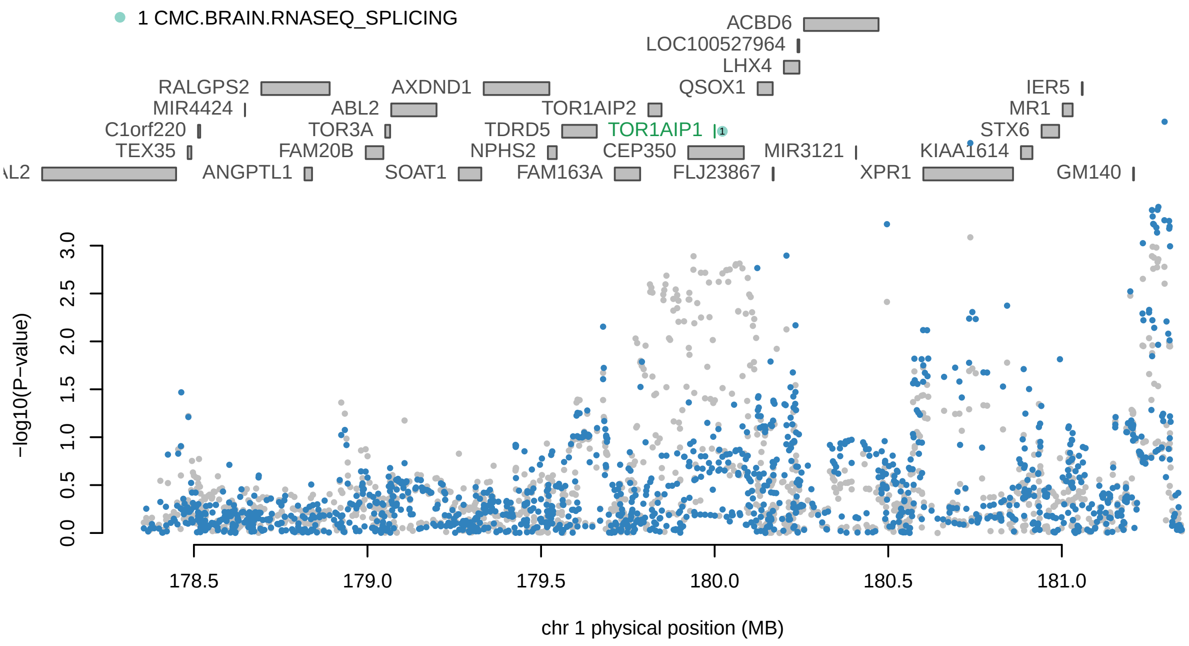

Supplementary Figure 14: FUSION plot of PD TWAS locus

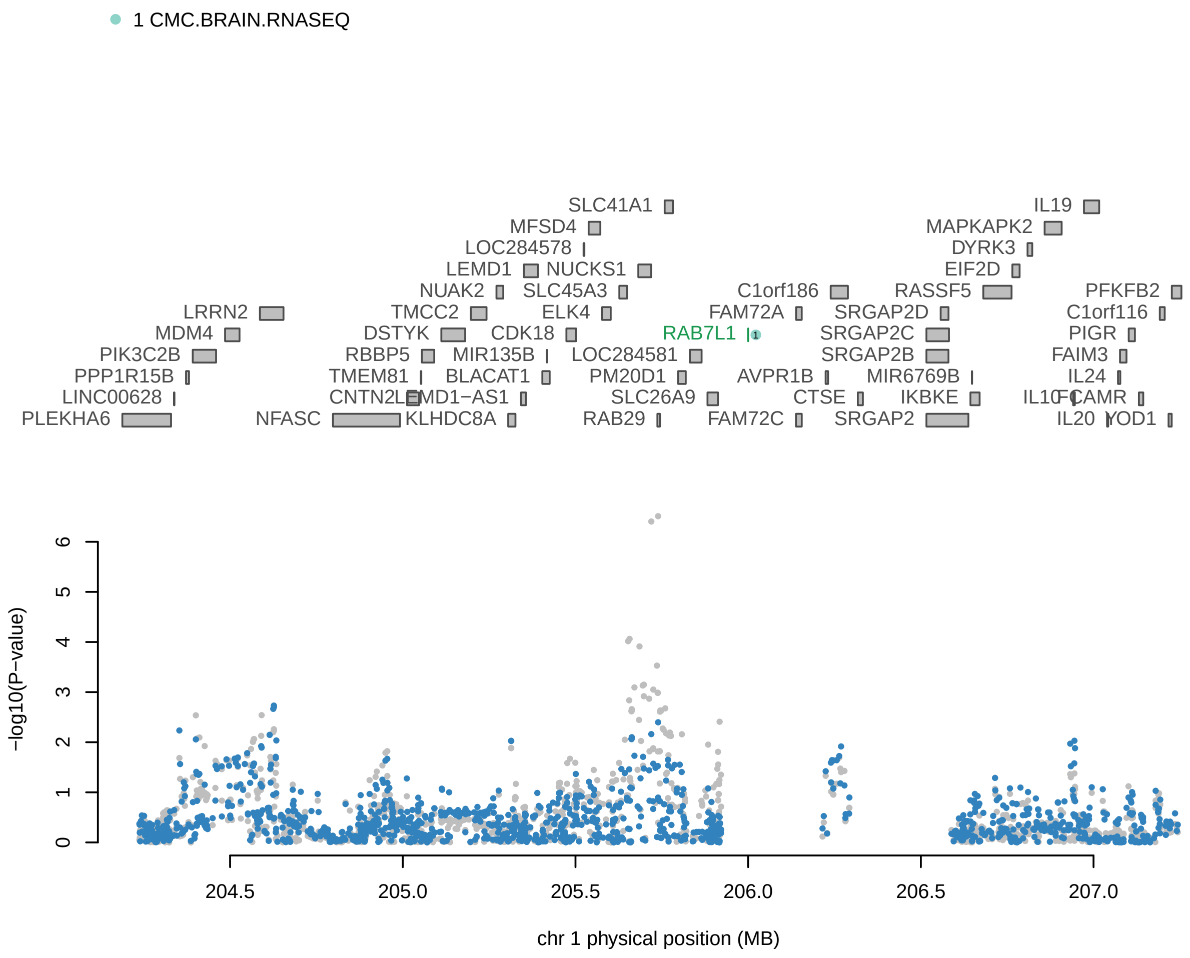

Supplementary Figure 15: FUSION plot of PD TWAS locus

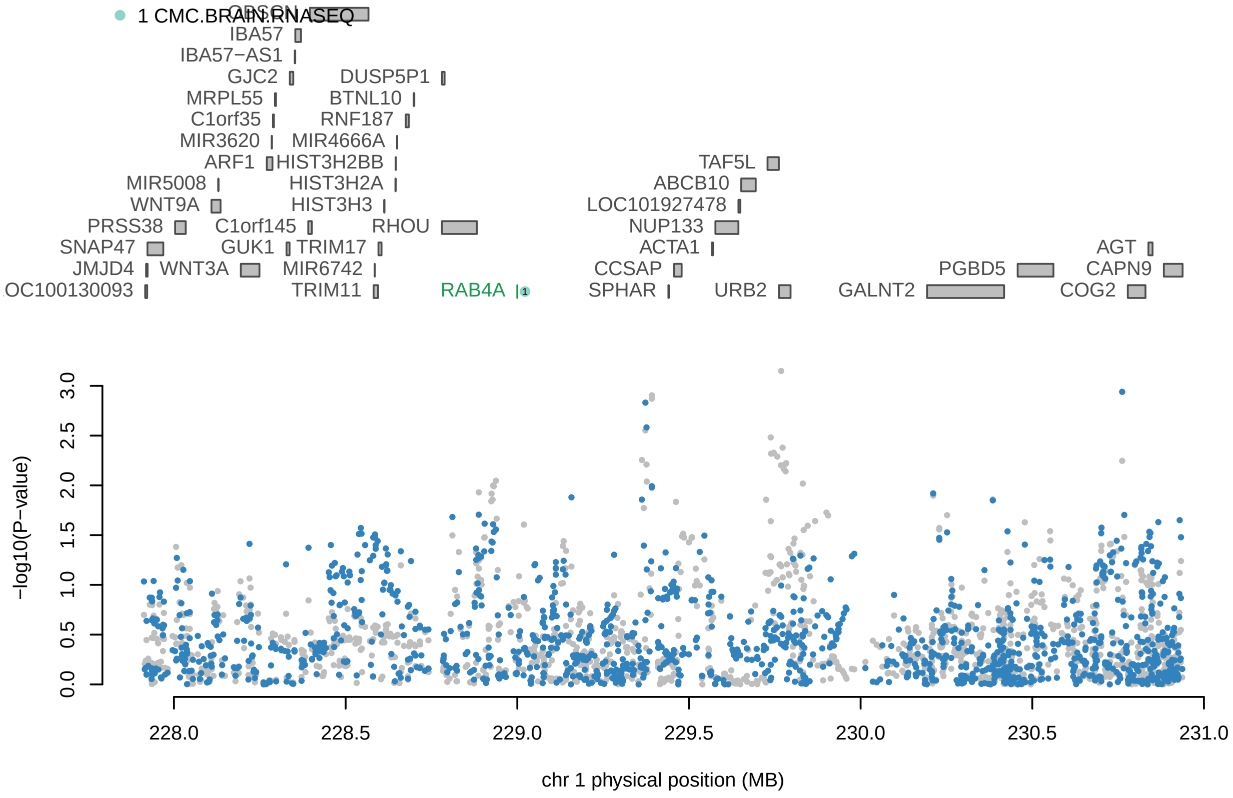

Supplementary Figure 16: FUSION plot of PD TWAS locus

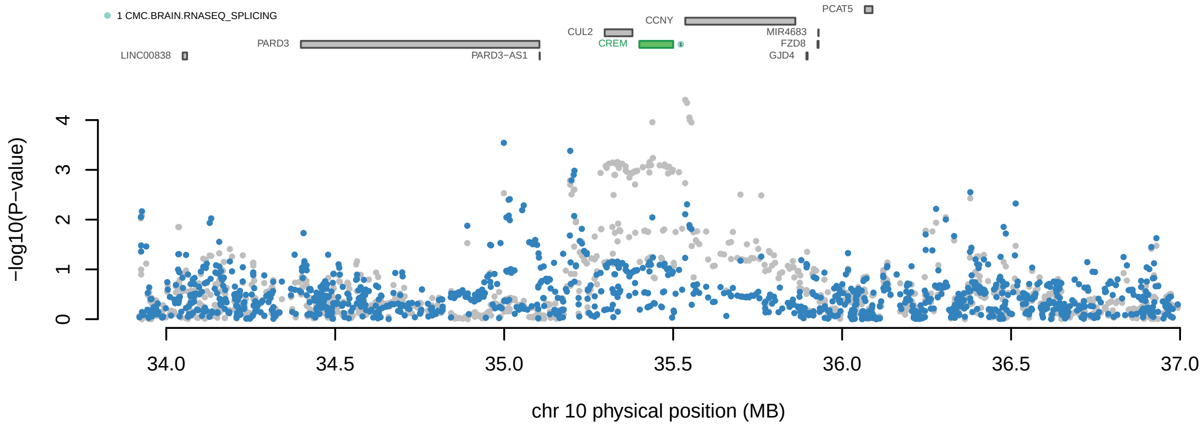

Supplementary Figure 17: FUSION plot of PD TWAS locus

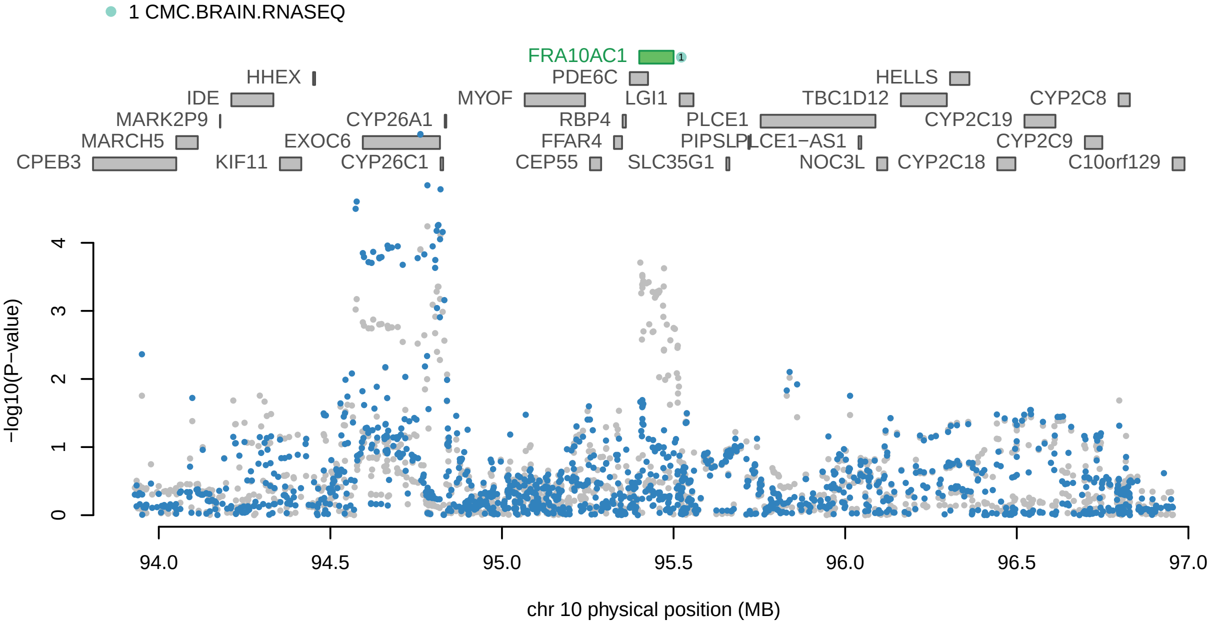

Supplementary Figure 18: FUSION plot of PD TWAS locus

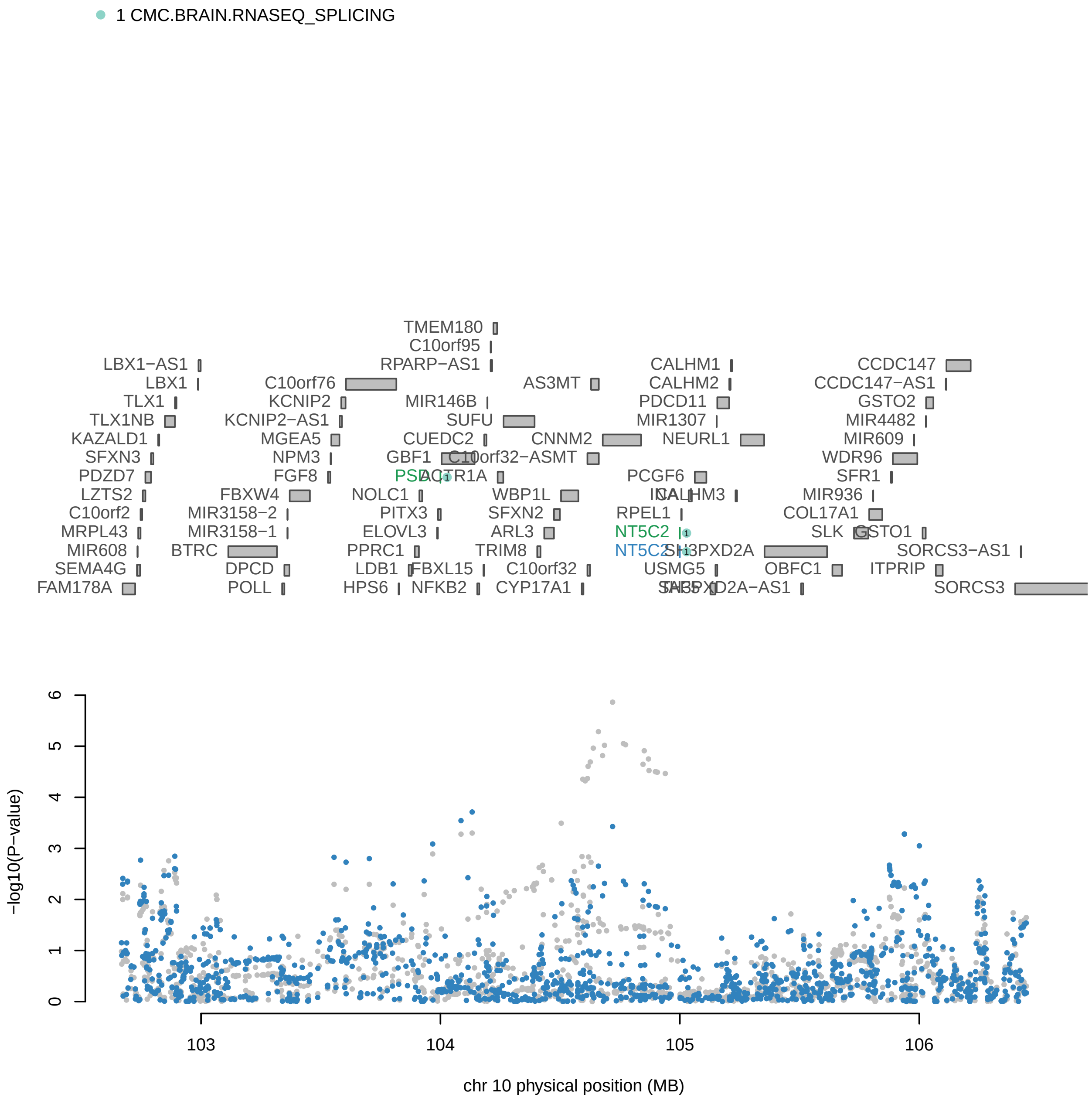

Supplementary Figure 19: FUSION plot of PD TWAS locus

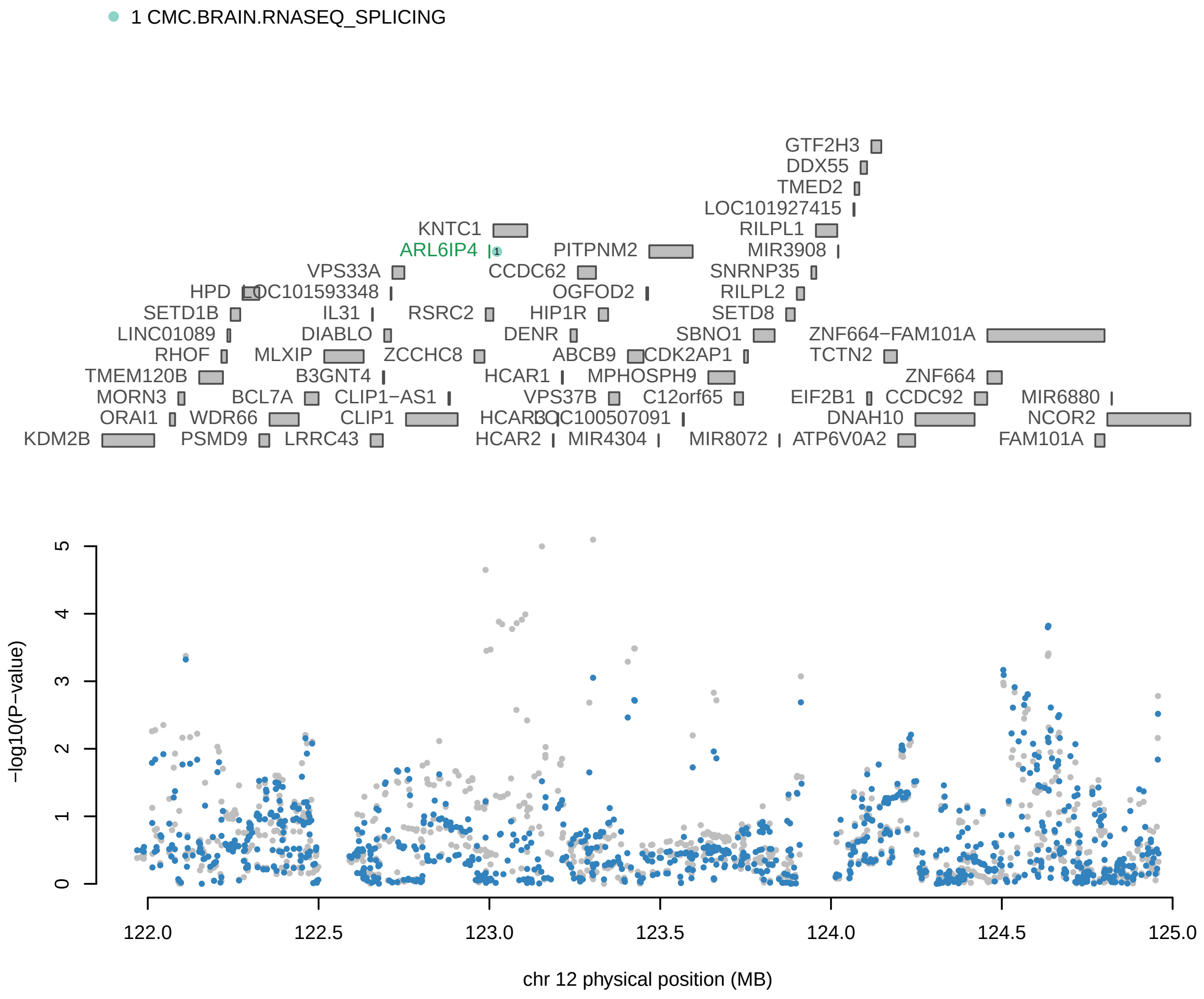

Supplementary Figure 20: FUSION plot of PD TWAS locus

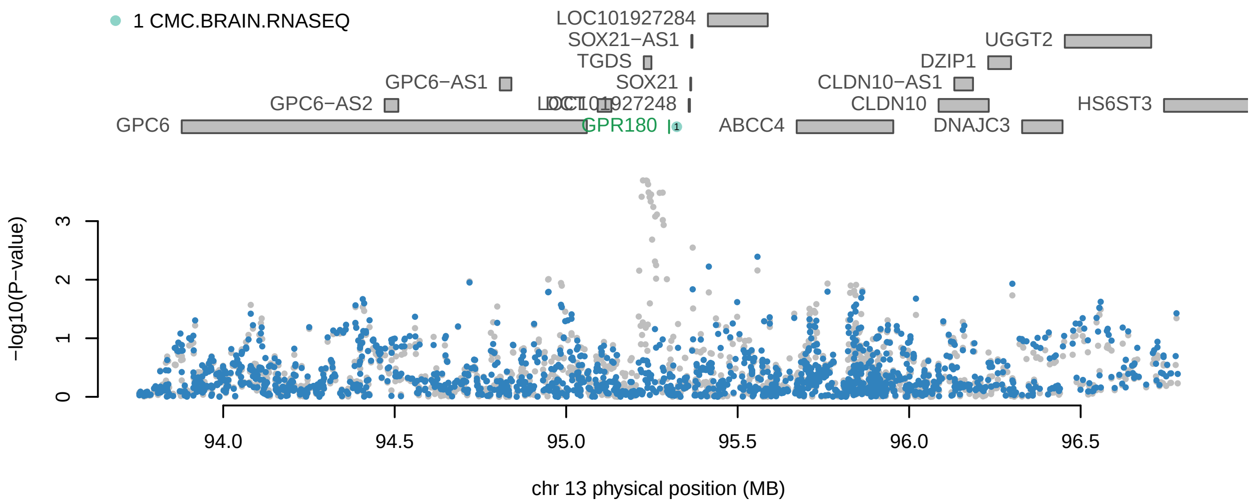

Supplementary Figure 21: FUSION plot of PD TWAS locus

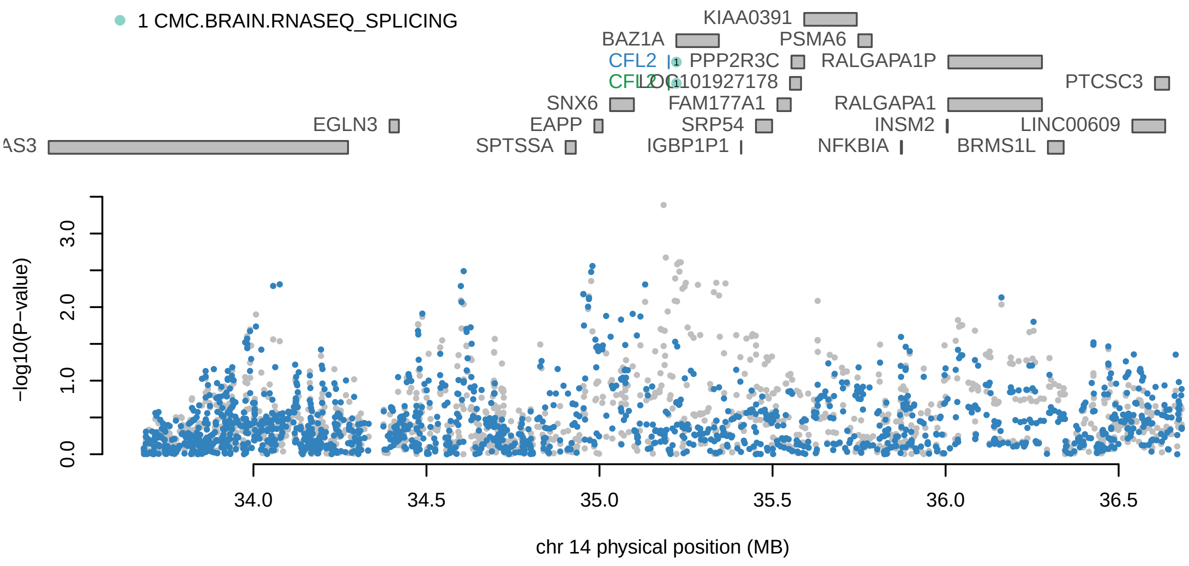

Supplementary Figure 22: FUSION plot of PD TWAS locus

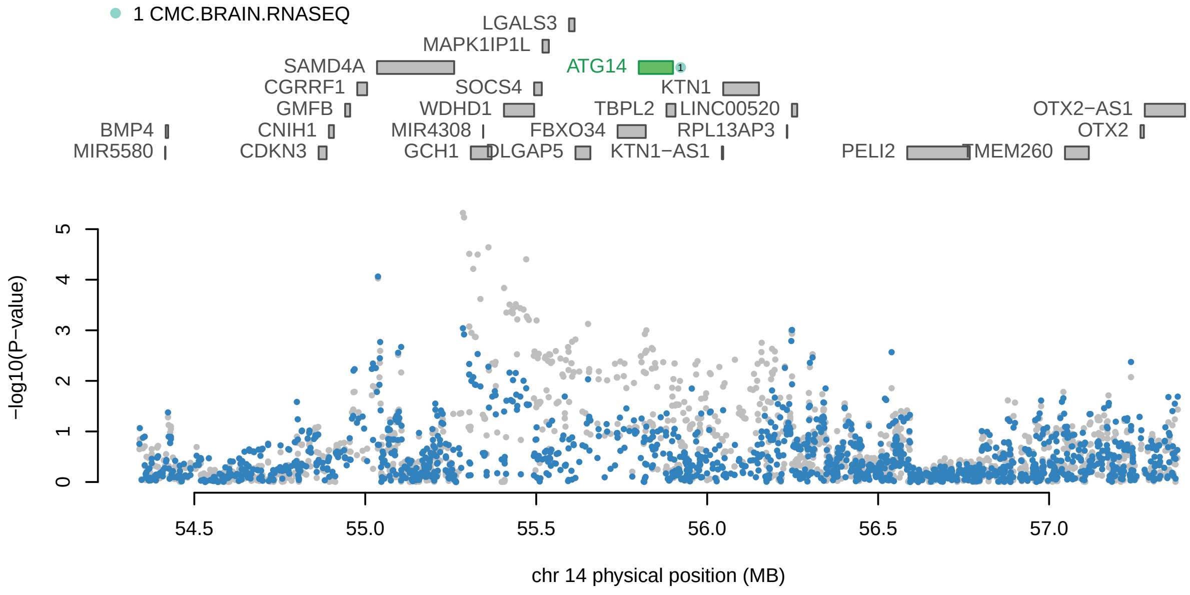

Supplementary Figure 23: FUSION plot of PD TWAS locus

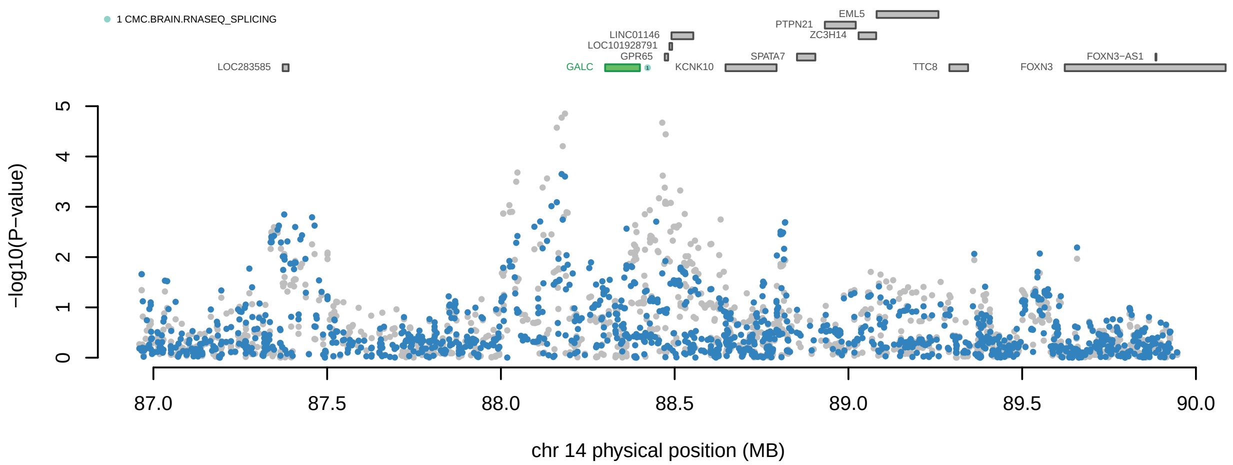

Supplementary Figure 24: FUSION plot of PD TWAS locus

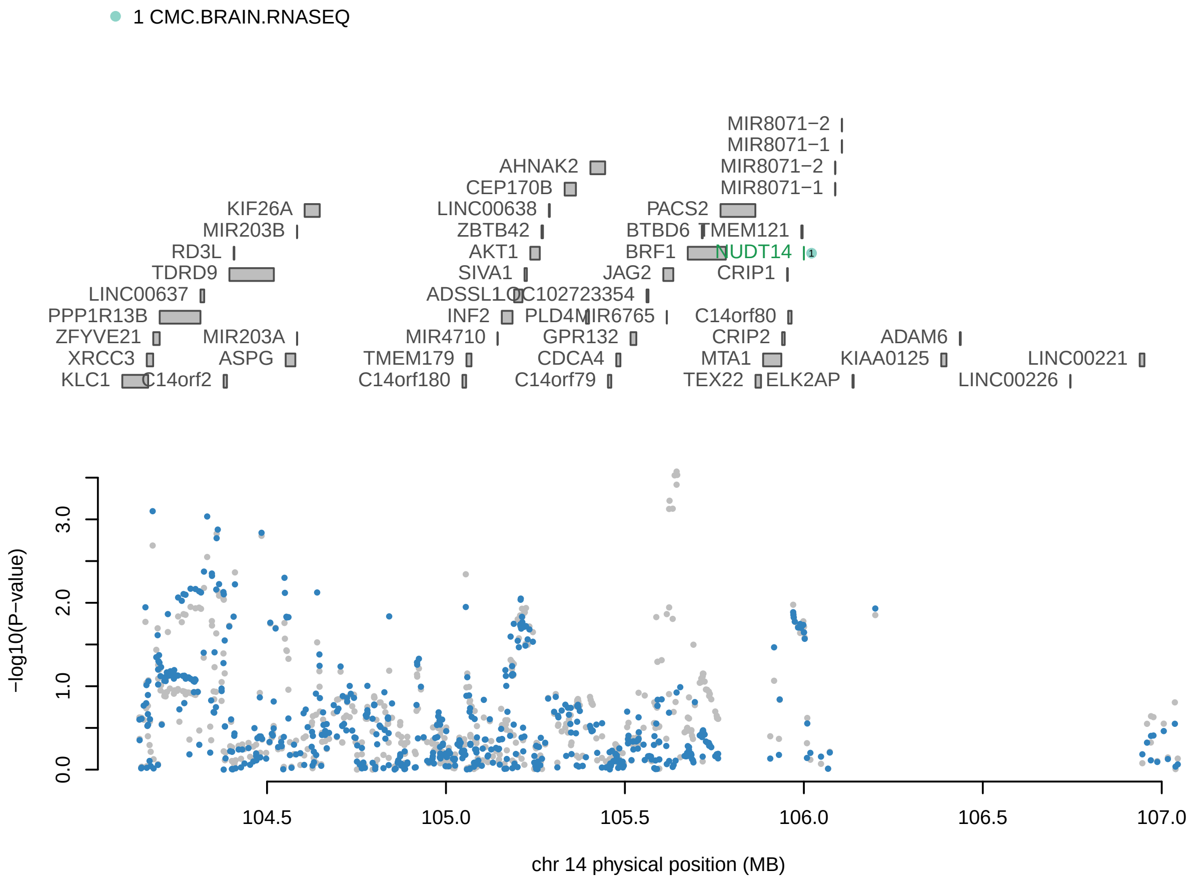

Supplementary Figure 25: FUSION plot of PD TWAS locus

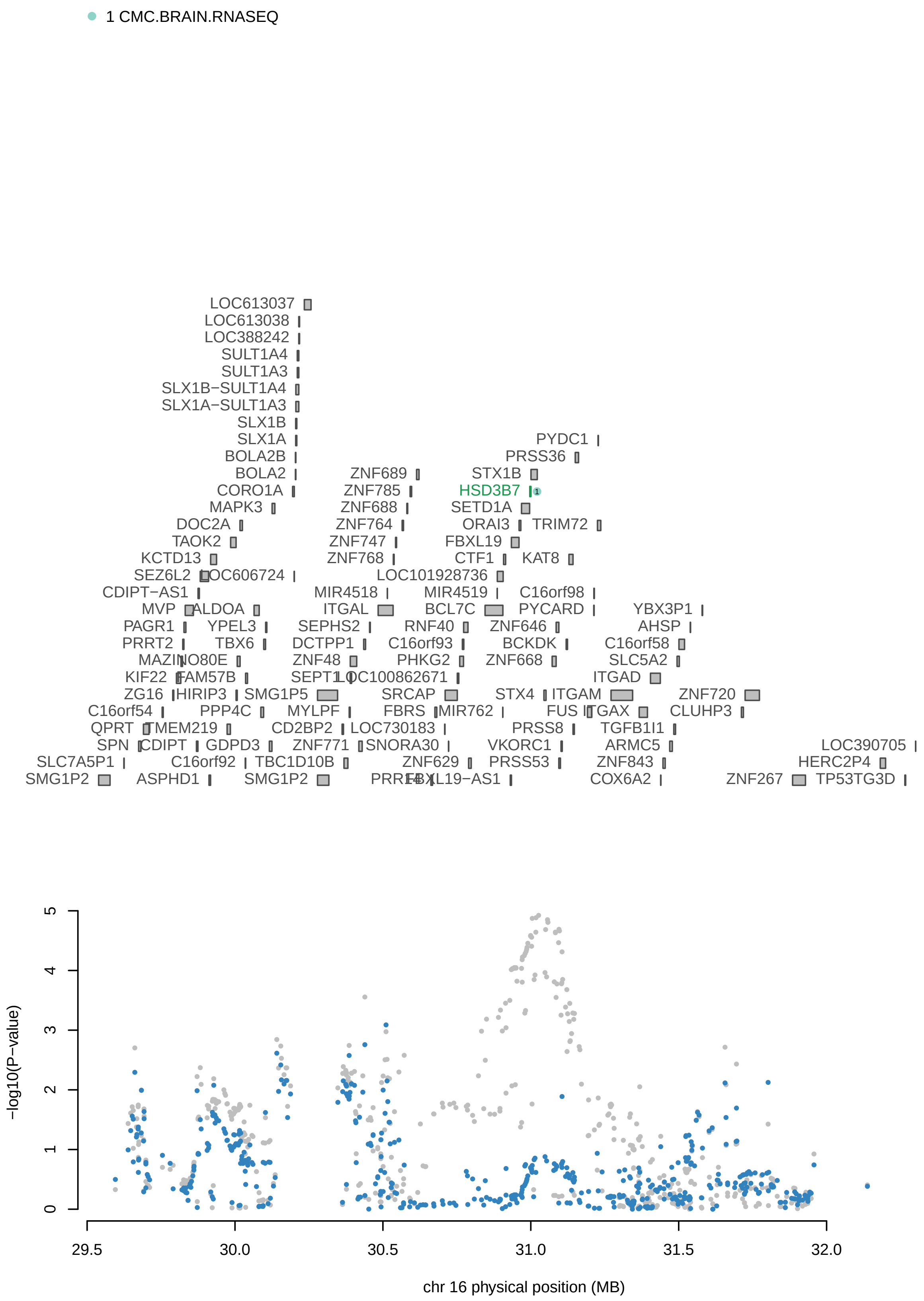

Supplementary Figure 26: FUSION plot of PD TWAS locus

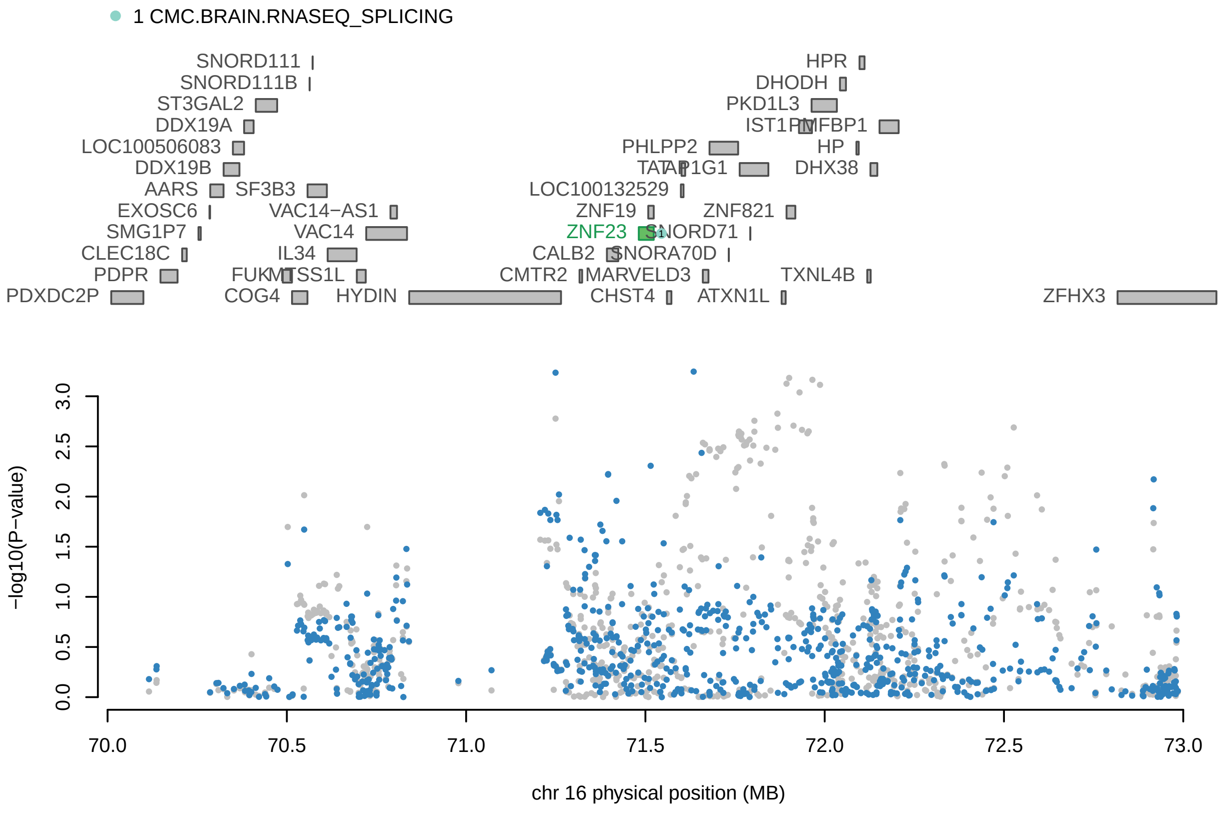

Supplementary Figure 27: FUSION plot of PD TWAS locus

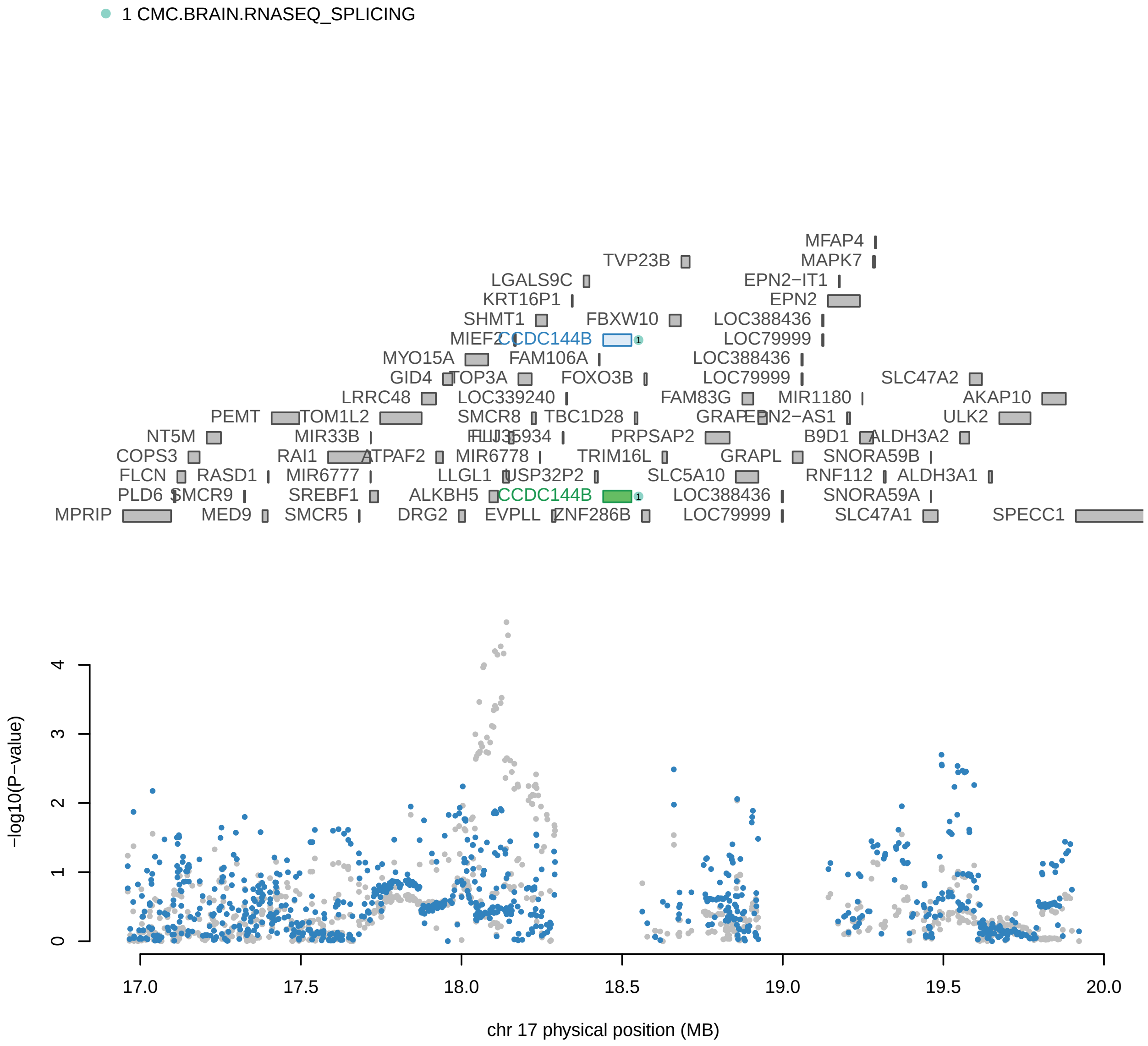

Supplementary Figure 28: FUSION plot of PD TWAS locus

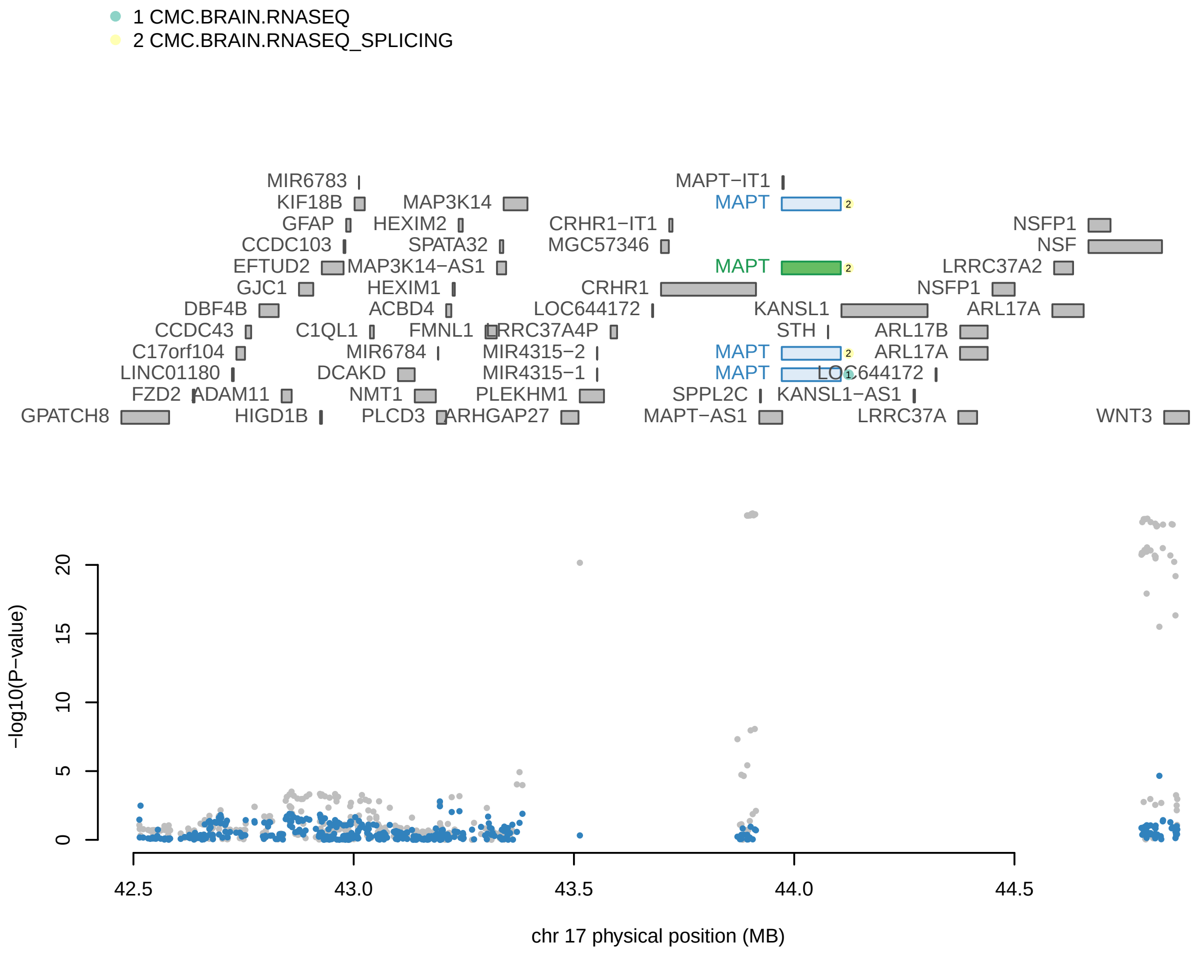

Supplementary Figure 29: FUSION plot of PD TWAS locus

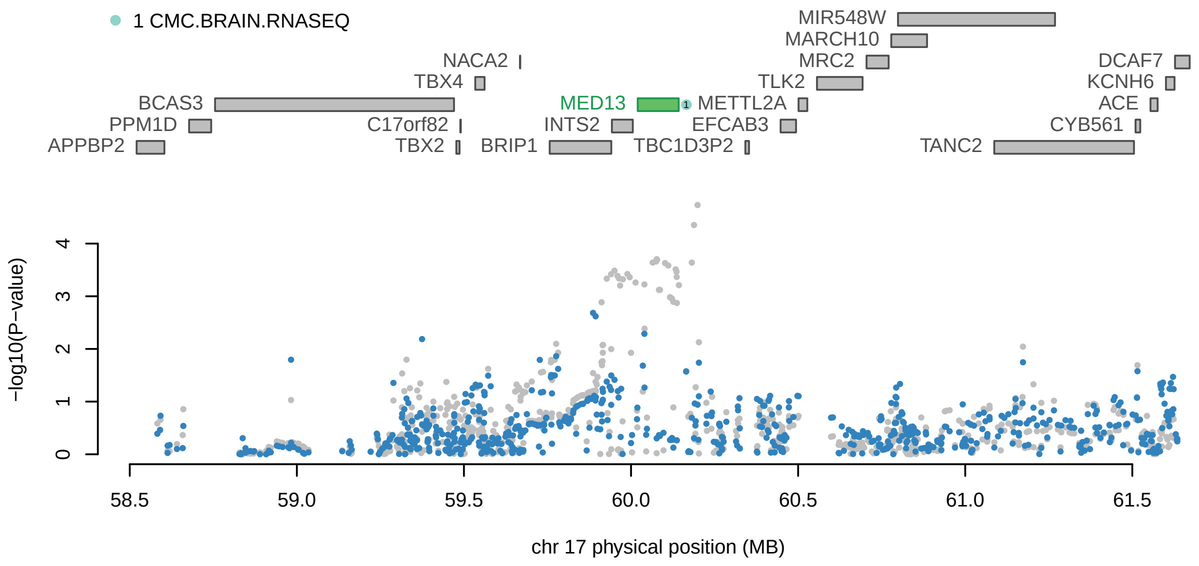

Supplementary Figure 30: FUSION plot of PD TWAS locus

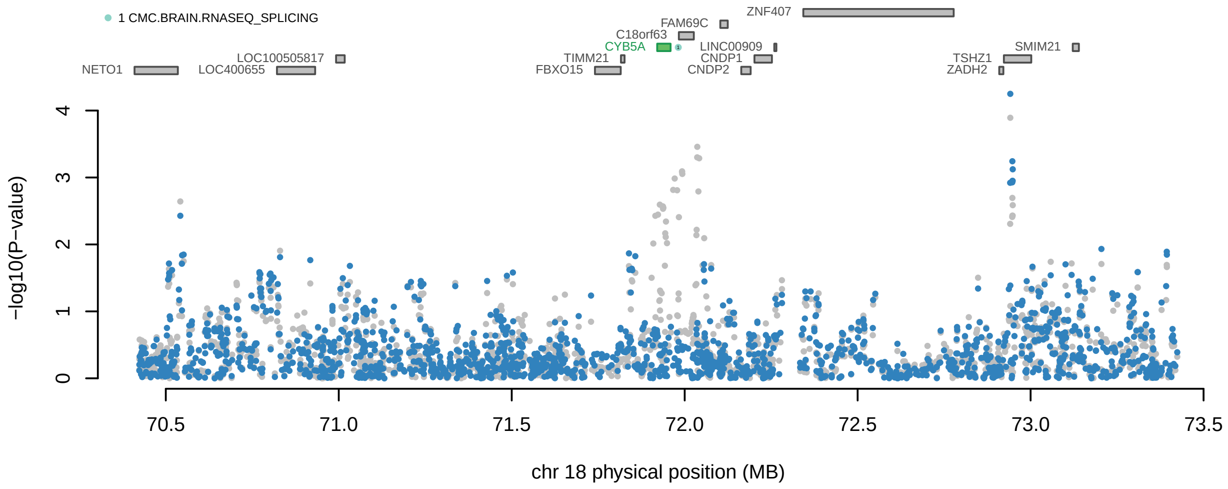

Supplementary Figure 31: FUSION plot of PD TWAS locus

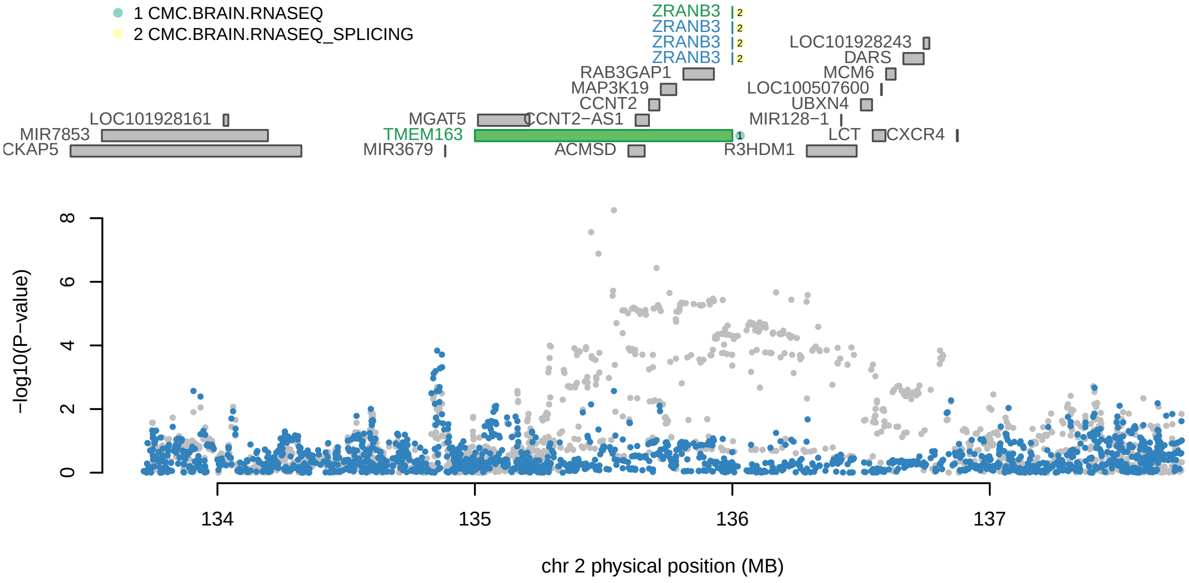

Supplementary Figure 32: FUSION plot of PD TWAS locus

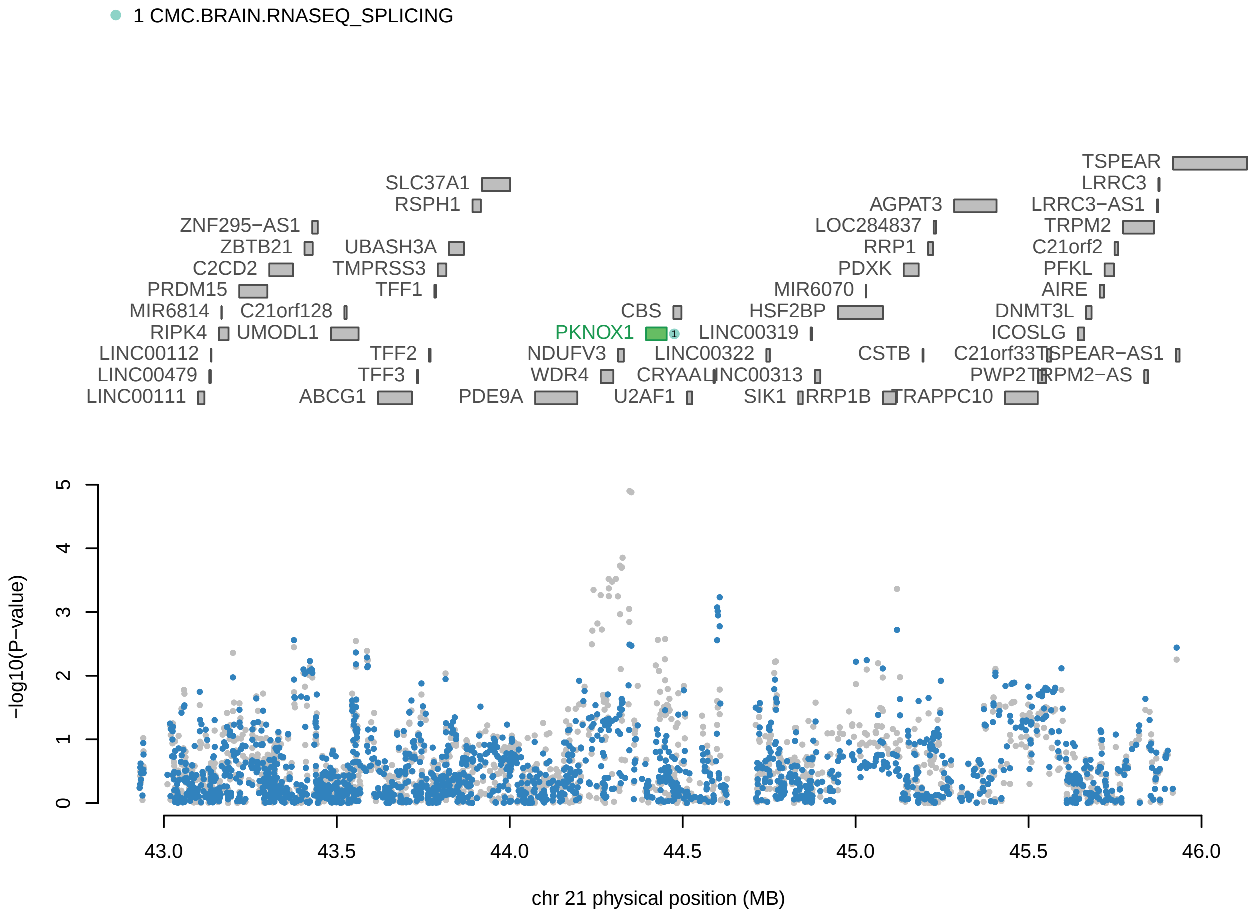

Supplementary Figure 33: FUSION plot of PD TWAS locus

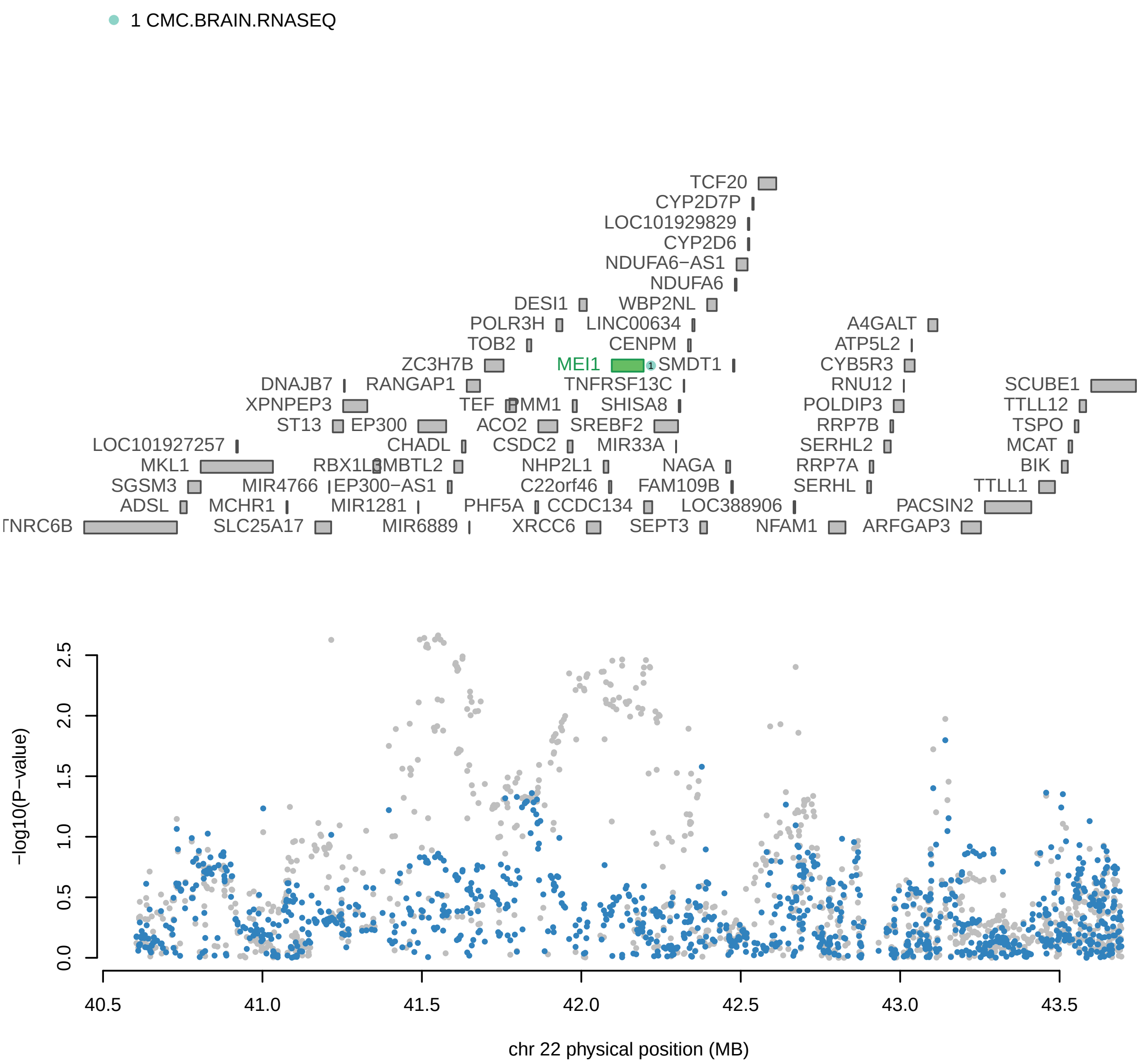

Supplementary Figure 34: FUSION plot of PD TWAS locus

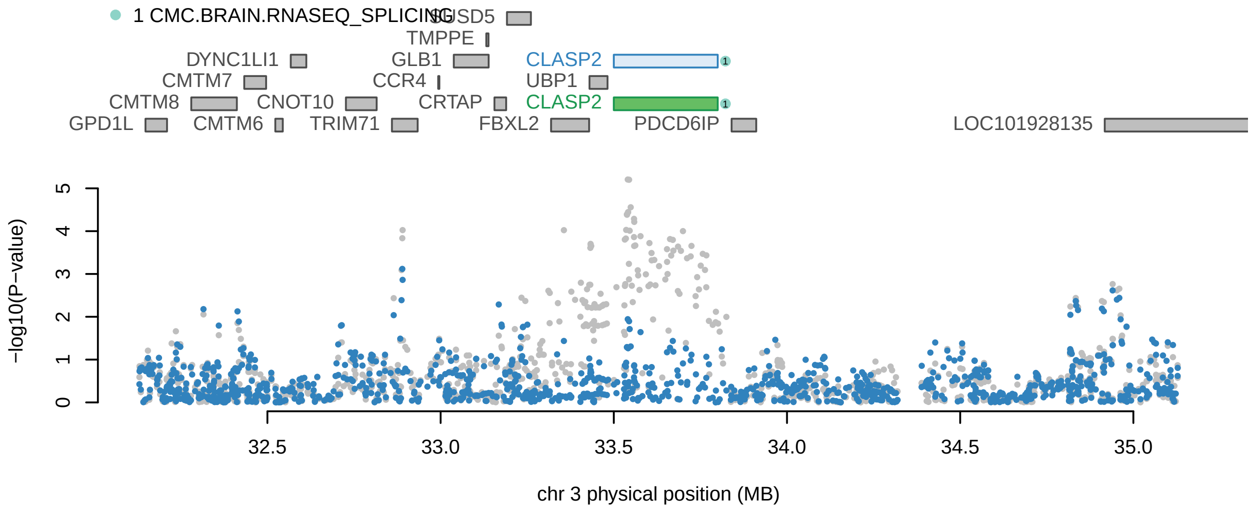

Supplementary Figure 35: FUSION plot of PD TWAS locus

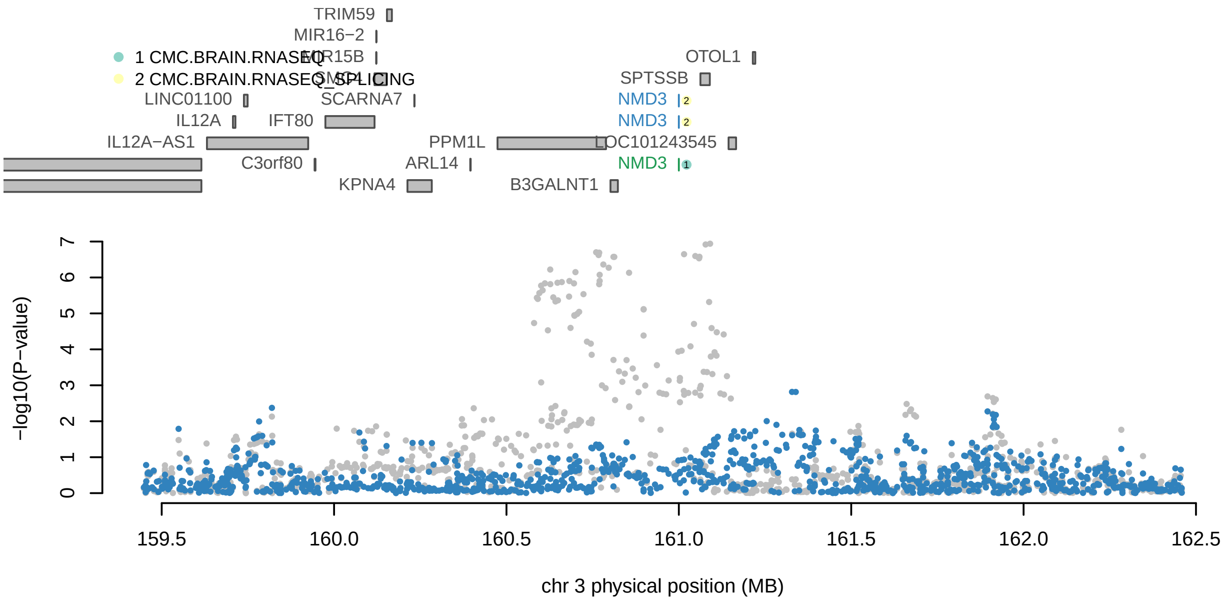

Supplementary Figure 36: FUSION plot of PD TWAS locus

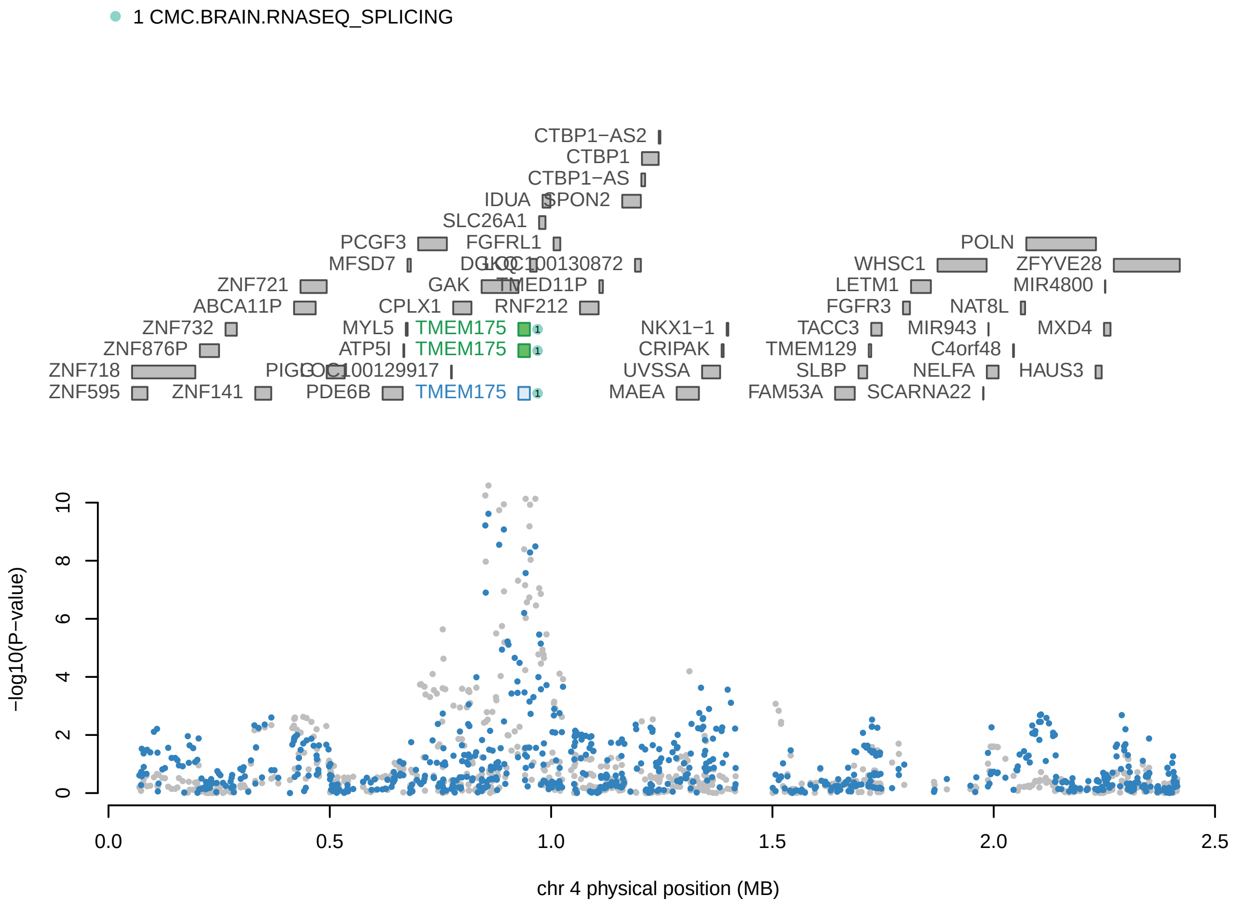

Supplementary Figure 37:FUSION plot of PD TWAS locus

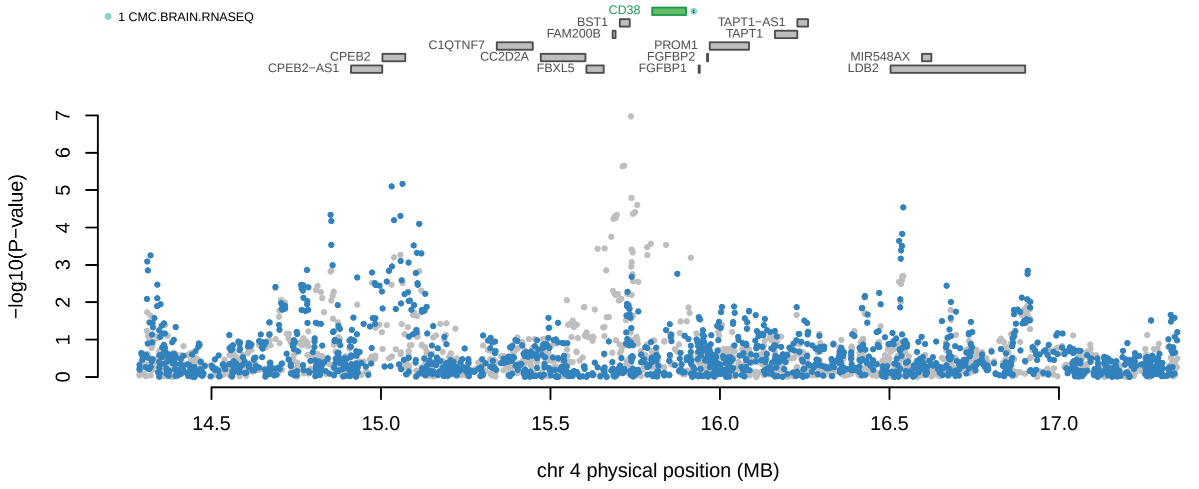

Supplementary Figure 38: FUSION plot of PD TWAS locus

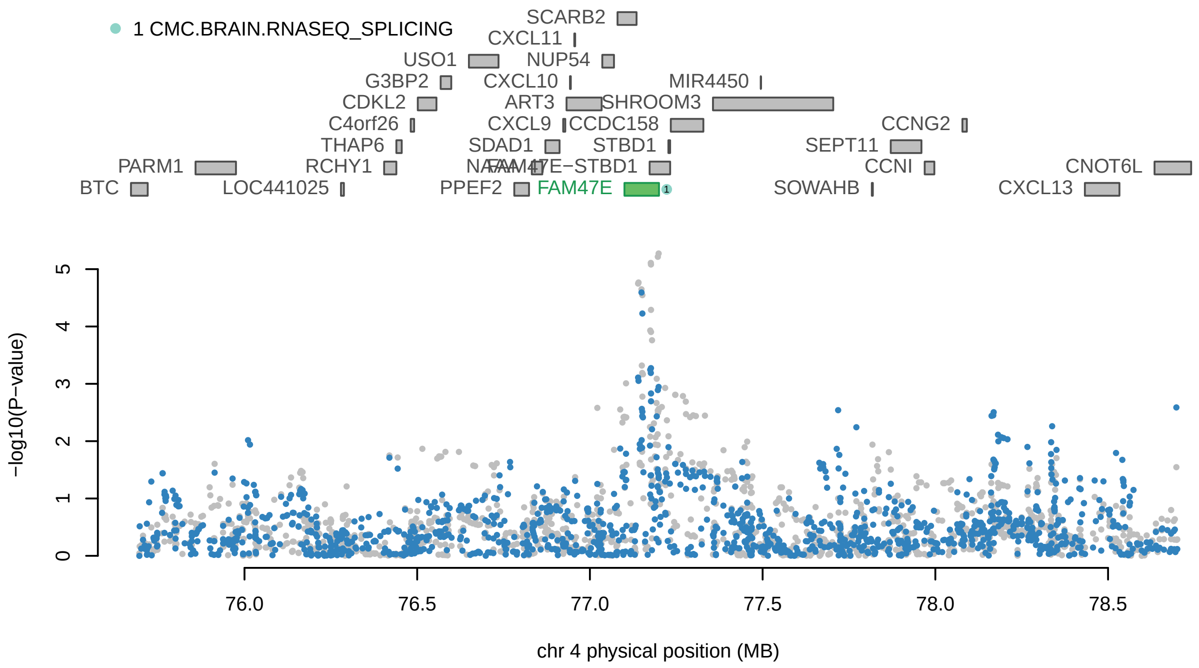

Supplementary Figure 39: FUSION plot of PD TWAS locus

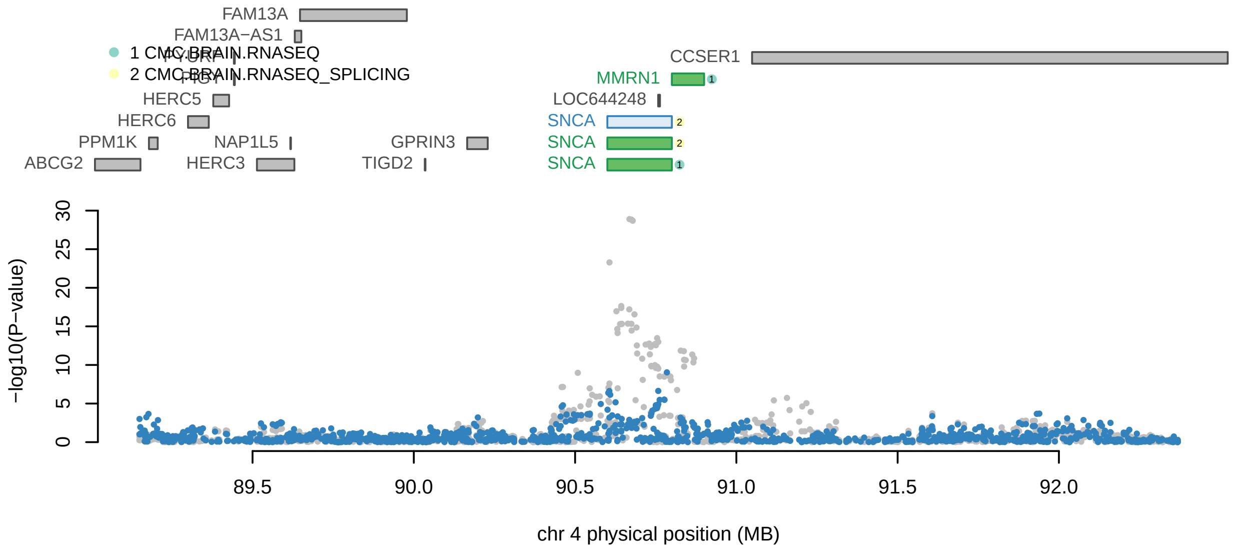

Supplementary Figure 40: FUSION plot of PD TWAS locus

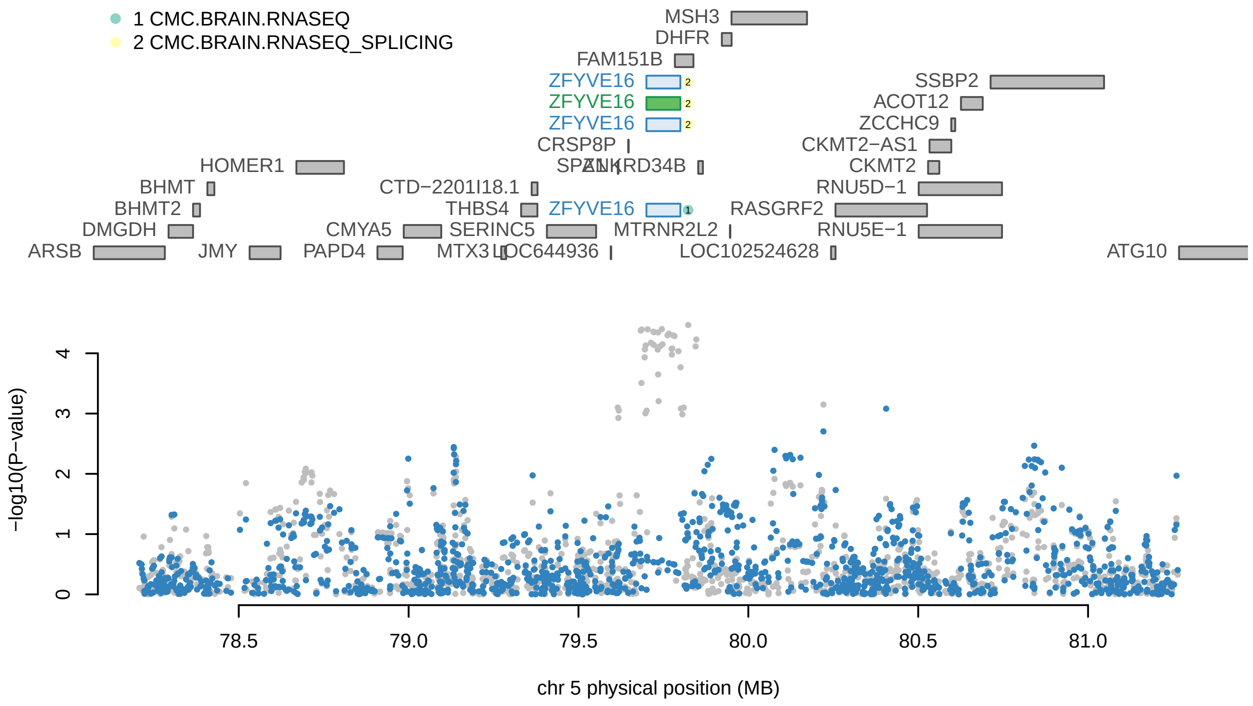

Supplementary Figure 41: FUSION plot of PD TWAS locus

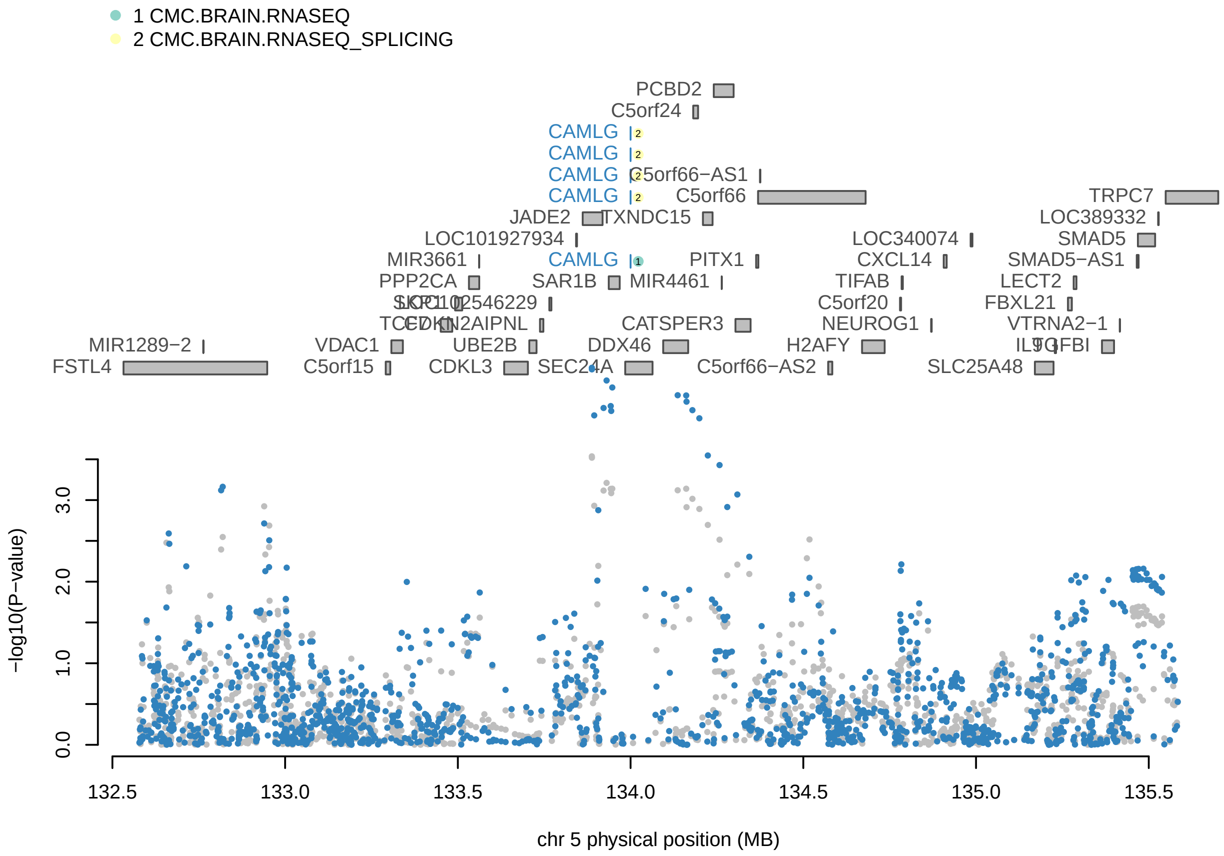

Supplementary Figure 42: FUSION plot of PD TWAS locus

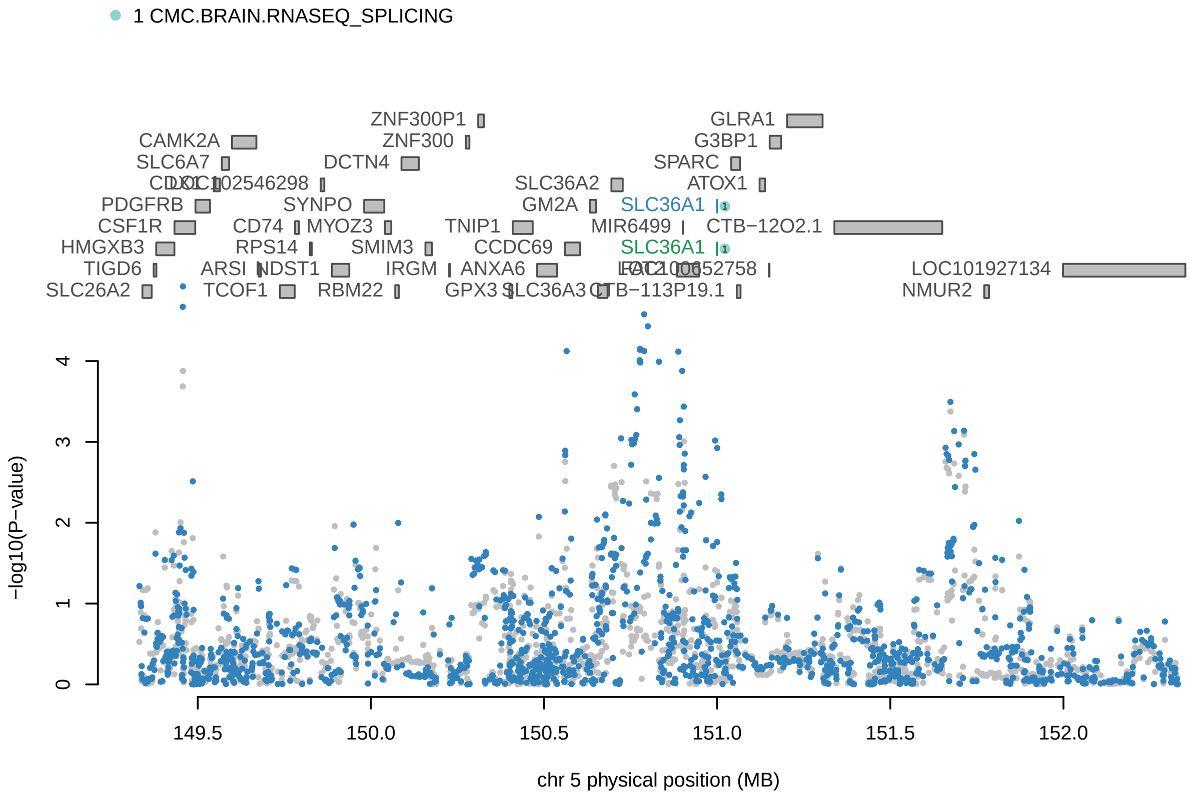

Supplementary Figure 43: FUSION plot of PD TWAS locus

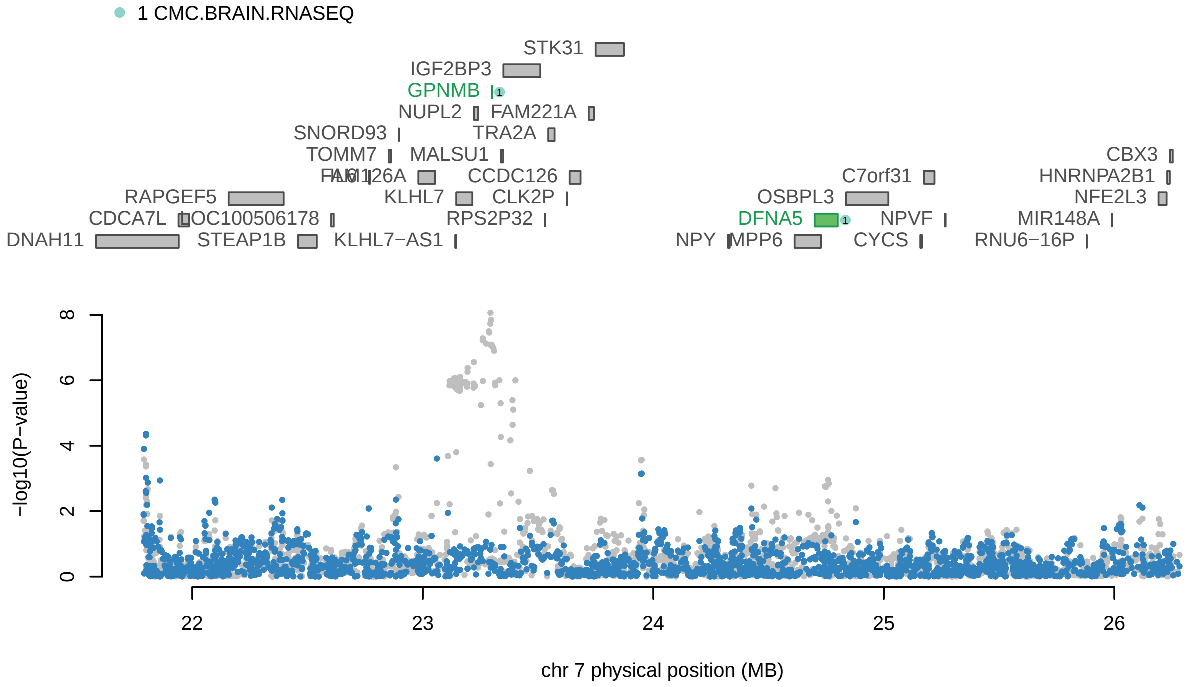

Supplementary Figure 44: FUSION plot of PD TWAS locus

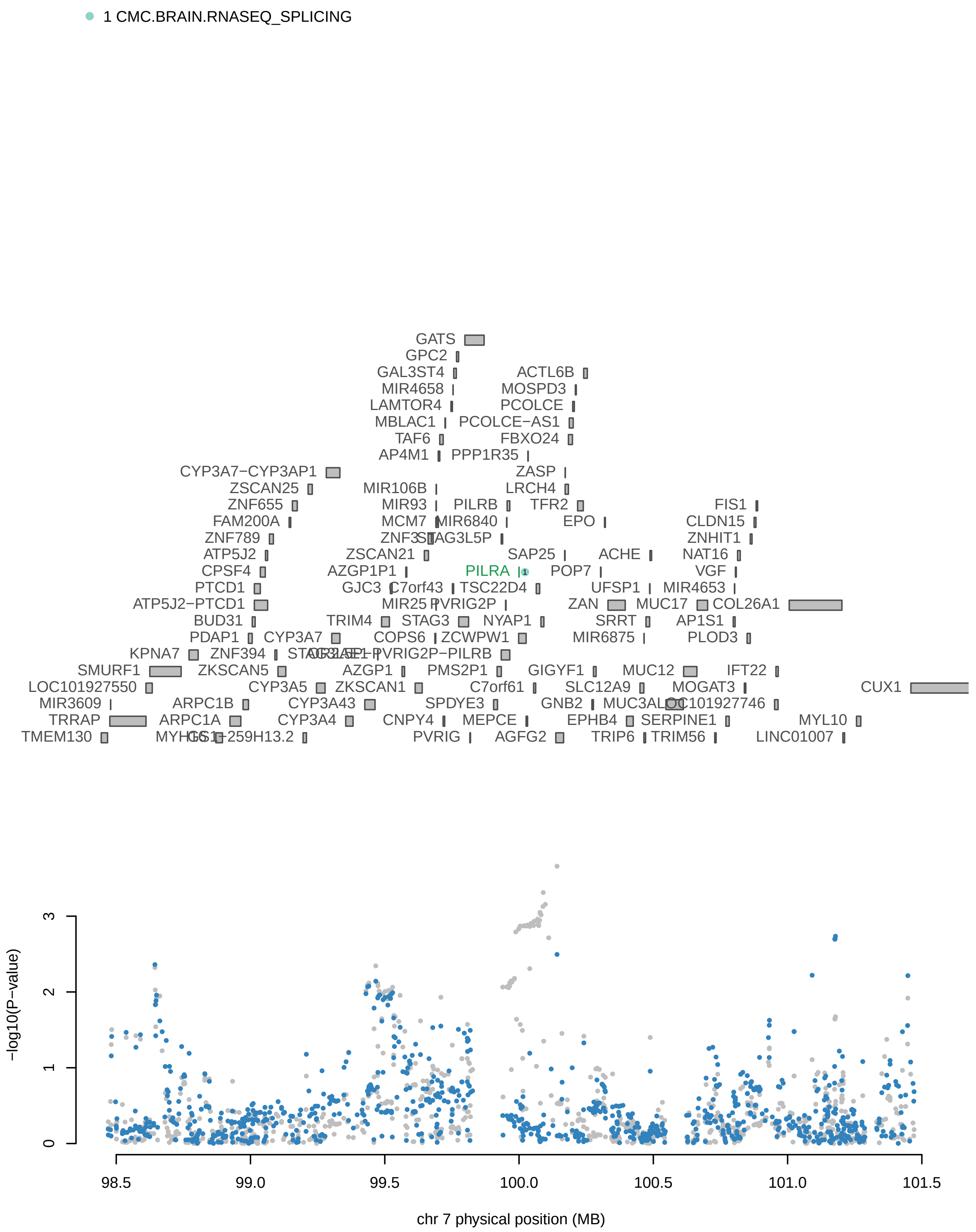

Supplementary Figure 45: FUSION plot of PD TWAS locus

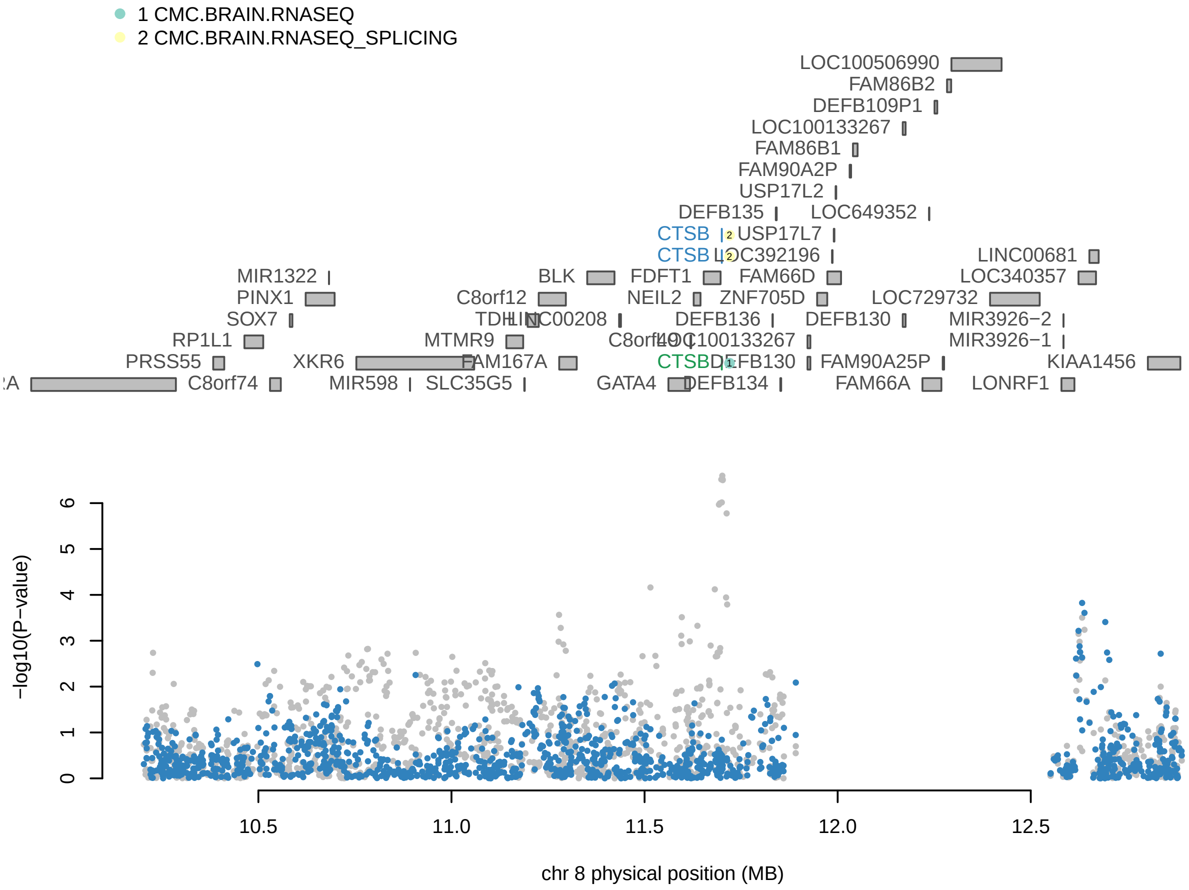

Supplementary Figure 46: FUSION plot of PD TWAS locus

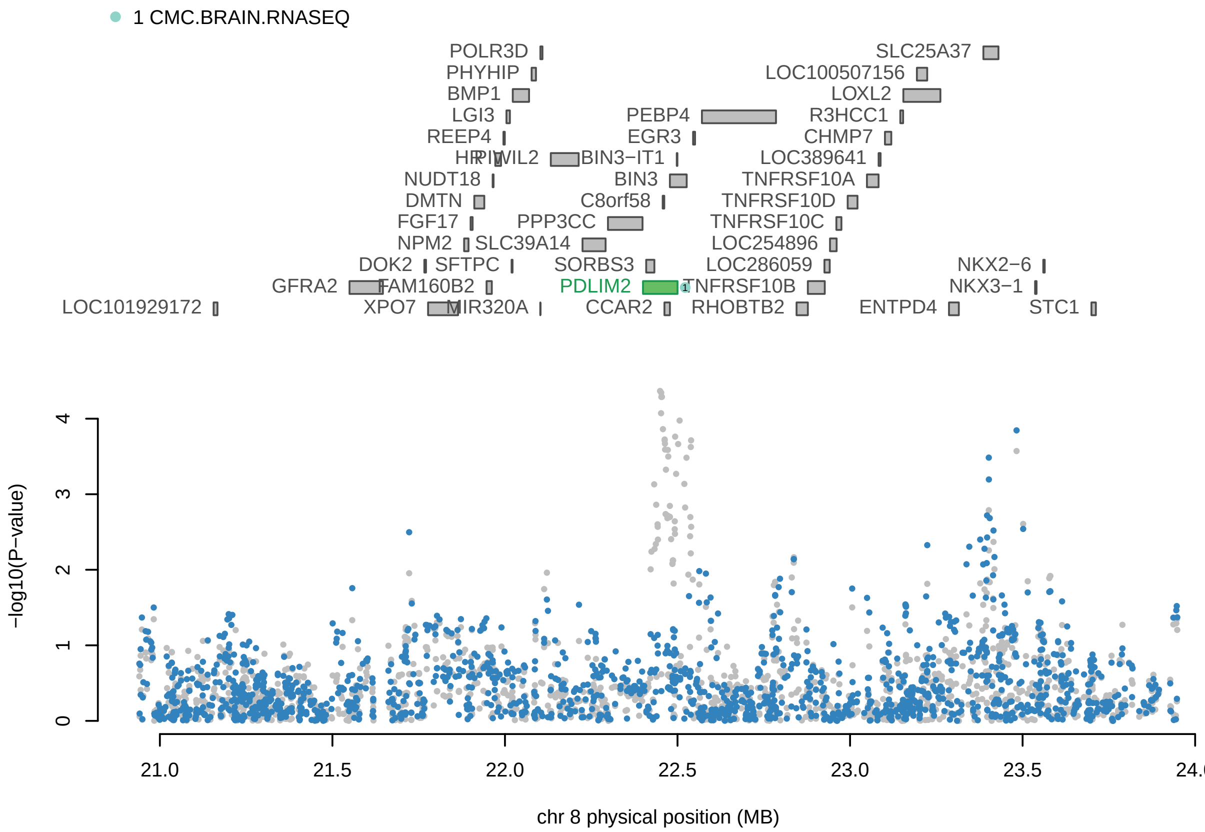

Supplementary Figure 47: FUSION plot of PD TWAS locus

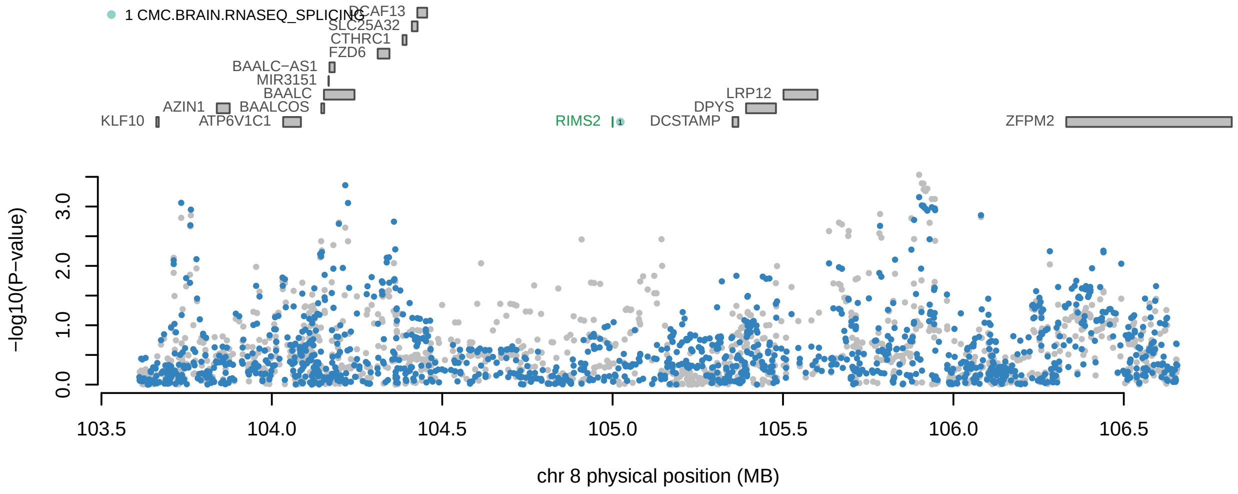

Supplementary Figure 48: FUSION plot of PD TWAS locus

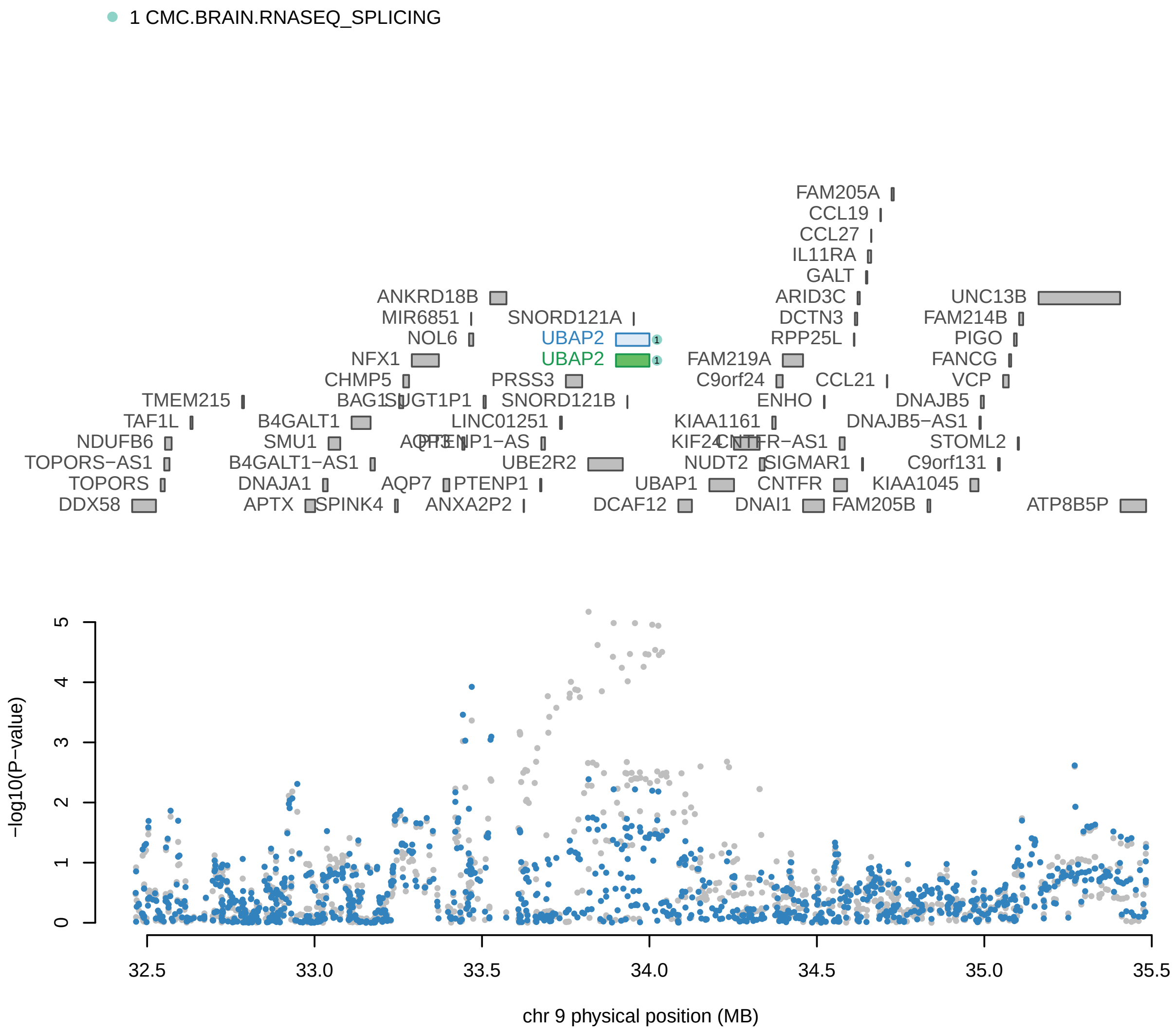

Supplementary Figure 49: FUSION plot of PD TWAS locus
