# Supplementary material for: Prioritizing Parkinson’s disease genes using population-scale transcriptomic data
Source: Nat Commun. 2019 Mar 1;10:994. doi: 10.1038/s41467-019-08912-9 (PMC6397174; doi:10.1038/s41467-019-08912-9)
Supplement: Supplementary file 3 — Description of Additional Supplementary Files [file 41467_2019_8912_MOESM3_ESM.pdf]

Description of Additional Supplementary Files

Supplementary Data 1

TWAS summary statistics for DLFPC splicing, gene expression and monocytes at FDR 0.05.
